# Pronouns Are as Sensitive to Structural Constraints as Reflexives in Early Processing: Evidence From Visual World Paradigm Eye-Tracking

**DOI:** 10.3389/fpsyg.2021.611466

**Published:** 2021-02-05

**Authors:** Chung-hye Han, Keir Moulton, Trevor Block, Holly Gendron, Sander Nederveen

**Affiliations:** ^1^Department of Linguistics, Simon Fraser University, Burnaby, BC, Canada; ^2^Department of Linguistics, University of Toronto, Toronto, ON, Canada; ^3^Department of Linguistics, University of British Columbia, Vancouver, BC, Canada

**Keywords:** binding theory, binding principle B, binding principle A, pronouns, reflexives, visual world paradigm eye-tracking

## Abstract

A number of studies in the extant literature report findings that suggest asymmetry in the way reflexive and pronoun anaphors are interpreted in the early stages of processing: that pronouns are less sensitive to structural constraints, as formulated by Binding Theory, than reflexives, in the initial antecedent retrieval process. However, in previous visual world paradigm eye-tracking studies, these conclusions were based on sentences that placed the critical anaphors within picture noun phrases or prepositional phrases, which have independently been shown not to neatly conform to the Binding Theory principles. We present results from a visual world paradigm eye-tracking experiment that show that when critical anaphors are placed in the indirect object position immediately following a verb as a recipient argument, pronoun and reflexive processing are equally sensitive to structural constraints.

## 1. Introduction

When a reader or listener encounters a pronoun or a reflexive anaphor, they are faced with the task of determining the referent of the anaphor. The interpretation of pronouns and reflexives is constrained by structural principles (Chomsky, 1981) and the question arises as to how and when these structural constraints are integrated into a reader or listener's understanding of reference. Reflexives require a local antecedent within the clause (Binding Principle A) while pronouns cannot resolve to a local antecedent (Binding Principle B). For instance, the reflexive *herself* in (1a) must corefer with *the girl*, the subject of the clause, and not with the subject of the embedded relative clause *the teacher*. The opposite holds for pronouns: as shown in (1b), the pronoun *her* may corefer with the subject of the embedded clause but not with the subject in its own clause.

(1)
  - The girl_1_ [that the teacher_2_ spoke to ] saw herself_1/*2_ in the mirror.
  - The girl_1_ [that the teacher_2_ spoke to ] saw her_*1/2_ in the store.

While these structural constraints hold of the ultimate interpretations these sentences may receive, questions arise about the time-course in processing these sentences. Much of the focus of psycholinguistic studies in this area examines the role of “interfering” antecedents, those phrases present in the sentence that are not ultimately grammatical antecedents but could potentially be mistakenly considered as such by comprehenders at various stages in processing. The key question is whether comprehenders ever consider these antecedents or whether the processor uses structural constraints like the Binding Theory to rule out such antecedents from the outset. Much of this research is framed in cue-based retrieval models with a content-addressable memory (Van Dyke and Lewis, 2003; Lewis and Vasishth, 2005; Lewis et al., 2006; Van Dyke and McElree, 2011). In such models, features on the anaphor are used as cues to retrieve an antecedent with matching features from memory. In principle, then, an anaphor could retrieve a structurally inappropriate antecedent that nonetheless bears matching morphological cues. In that case, interfering noun phrases could have either ameliorating or deleterious effects on processing. Alternatively, structural constraints could serve to filter out such antecedents, or weight morphological cues less than structural factors. A number of studies, using both behavioral measures and neurological responses, point in favor of prioritizing structural constraints in initial processing (Nicol and Swinney, 1989; Clifton et al., 1999; Harris et al., 2000; Xiang et al., 2009; Dillon et al., 2013; Chow et al., 2014; Cunnings and Sturt, 2014). However, the experimental record is far from uniform and the interaction between morphological and structural cues remains an active area of investigation (Parker and Phillips, 2017; Jäger et al., 2020).

There are at least two types of interference effects that are relevant to these questions. One is the case of *facilitatory interference*, where grammatically inappropriate but feature-matching antecedents ameliorate the processing of ungrammatical sentences. Such facilitatory interference effects have been documented for cases of agreement mismatches (Wagers et al., 2009). Whether anaphoric dependencies also exhibit facilitatory interference is under active investigation. In the case of reflexives, facilitatory interference is expected to manifest as faster reading times for reflexives in sentences such as (2a) compared to (2b) (from Dillon et al., 2013). While both are ungrammatical, faster reading times for the reflexive in (2a) would be taken to indicate that the interfering noun phrase *professional trainers* (the “inaccessible antecedent”) is at least momentarily taken by the processor as a suitable antecedent, whereas in (2b) no such feature matching antecedent is available.

(2)
  - The amateur body builder who worked with the **professional trainers** amazingly injured **themselves** on the lightest weights.
  - The amateur body builder who worked with the **professional trainer** amazingly injured **themselves** on the lightest weights.

In eye-tracking while reading studies, Dillon et al. (2013) found no evidence of facilitatory interference in the case of reflexive dependencies, in contrast to agreement dependencies which did show the effect. They took these findings to show that anaphoric and agreement dependencies make use of different retrieval mechanisms, such that in reflexive dependencies, structural cues gate retrieval to only those noun phrases compatible with the grammar. In a large-scale replication of Dillon et al. (2013), Jäger et al. (2020) found evidence for interference but only in reading measures commonly associated with later processing. This finding potentially bears out Sturt's (2003) claim that Binding Theory constrains initial retrieval but comprehenders consider grammatically unavailable antecedents later in processing. Facilitatory interference effects are also reported in Patil et al. (2016) and Parker and Phillips (2017). The latter found facilitatory effects only when the structurally appropriate antecedent mismatched the reflexive in two features (e.g., number *and* gender), not just one. They conclude that structural factors are more heavily weighted than morphological cues, but the latter do have an influence.

In the case of pronominal anaphora, Chow et al. (2014) find no evidence for facilitatory interference using both self-paced reading and eye-tracking while reading. They do find that in ungrammatical sentences a matching but structurally ungrammatical antecedent can slow reading times (see Sturt, 2003). Chow et al. (2014) interpret these results as evidence for the *Simultaneous Hypothesis*, whereby both structural and non-structural constraints (e.g., morphological cues) determine which antecedents are subject to retrieval.

Another type of interference effect is *inhibitory interference* (Dillon, 2011), where a processing cost is incurred when there is feature match with more than one antecedent—what we will call “double match” situations. One such instance is illustrated in (3a) where both the Binding Theory appropriate antecedent and an interfering, ungrammatical antecedent match the gender of the reflexive.

(3)
  - The amateur body builder who worked with the **professional trainer** amazingly injured **himself** on the lightest weights.
  - The amateur body builder who worked with the **professional trainers** amazingly injured **himself** on the lightest weights.

Cue-based retrieval models make the very clear prediction (see, e.g., Jäger et al. 2020) that such ‘cue overload’ in (3a) will give rise to processing difficulty compared to (3b), assuming that structural constraints do not entirely remove the interfering noun phrase as a candidate for retrieval. Unlike Dillon et al. (2013), Jäger et al. (2020) did find evidence for inhibitory interference in such configurations with reflexives; there were a greater number of first-pass regressions from the reflexive in (3a) compared to (3b).

Badecker and Straub (2002) report evidence of inhibitory interference in the case of pronominal anaphora. Among the types of sentences they tested were “double match” configurations as in (4a) (also referred to as “multiple match”). The structurally unavailable noun phrase *Bill* matches the masculine morphological cue of the pronoun. Badecker and Straub (2002) found processing costs in this condition compared to the condition in which there is only a single match with the structurally appropriate antecedent (4b).

(4)
  - Double match
    John thought that Bill owed him another chance to solve the problem.
  - Single match
    John thought that Beth owed him another chance to solve the problem.

A multiple or double match effect like this suggests structural constraints do not rule out retrieval of matching but grammatically inappropriate antecedents. However, Chow et al. (2014) failed to replicate the effect across five experiments, employing both self-paced reading and eye-tracking while reading methods, supporting their *Simultaneous Hypothesis*. Chow et al. (2014) also manipulated the degree of similarity of the competitor antecedents in terms of noun phrase type (proper vs. common vs. quantified noun), a factor that by definition affects similarity-based interference (Gordon et al., 2001). They found no inhibitory effect even when both competitor noun phrases were identical in type.

While the studies cited above collected reading time data, Runner and Head (2014) investigated double match configurations like those in (4) using the Visual World Paradigm (VWP). They examined processing of both reflexives and pronominal anaphora. Participants in their VWP eye-tracking study listened to auditory stimuli such as (5), which manipulated whether the target anaphor was a pronoun or a reflexive and whether an inaccessible antecedent matched the anaphor in gender or not. Each sentence contained two potential antecedents for the anaphor, a structurally accessible antecedent and a structurally inaccessible antecedent. For reflexives, the structurally accessible antecedent is the matrix subject [*the pharmacist*], and the structurally inaccessible antecedent is the subject in the relative clause (*Molly* or *Darrin*). For pronouns, the structurally accessible antecedent is the subject of the relative clause (*Molly*), and the structurally inaccessible antecedent is the matrix subject (*the pharmacist (m/f)*). One of the antecedents was instantiated as a gender-specific proper name, and the other as a gender-neutral occupation (pharmacist). Gender of the occupation was manipulated visually, using, for example, a picture of a male or a female pharmacist.

(5)
  - Reflexive
    The pharmacist(f) [that Molly/Darrin met] drove herself to the party.
  - Pronoun
    The pharmacist(m/f) [that Molly met] drove her to the party.

These sentences were presented auditorily while participants viewed a screen which displayed four pictures that represented the character of the proper name mentioned in the sentence, a distractor character not mentioned in the sentence, the occupation mentioned in the sentence, and a distractor occupation not mentioned in the sentence. If comprehenders consider gender-matched inaccessible antecedents in processing anaphora (either reflexives or pronouns), then there should be more looks in the double match condition to the inaccessible referent than in the single match condition. Runner and Head (2014) found that for both reflexives and pronouns, there were more looks to the inaccessible antecedent in the double match condition than in the single match condition. Moreover, they found that in the double match condition pronouns exhibited a higher amount of looks to the inaccessible antecedent in comparison to reflexives. While the finding of double match effects for both pronouns and reflexives is in line with the findings of Badecker and Straub (2002) and Jäger et al. (2020) in reading studies, the finding that pronouns showed a bigger double match effect than reflexives may be confounded by the discourse prominence of inaccessible antecedents. In all stimuli used in Runner and Head (2014) study, the inaccessible antecedent for the reflexive was located in a relative clause and the inaccessible antecedent for the pronoun was the matrix clause subject. Clausal subjects c-command target pronouns and have much higher discourse-prominence than non-commanding expressions within embedded relative clauses, and so it is likely that they attract more attention, resulting in more looks. Moreover, the gender manipulation of the inaccessible antecedent differed between pronouns and reflexives. In the pronoun conditions, participants had to determine the gender of the inaccessible antecedent through visual inspection of the depicted character and interpretation of their gender expression (hair, clothing). In contrast, the gender of the inaccessible antecedents in the reflexive condition is encoded linguistically by a gendered name. We do not know what effects this difference in the stimuli may have, although the effort required to visually interpret gender expression could make gender more salient in the pronoun cases.

The stimuli in the VWP study in Clackson et al. (2011) avoid these potential confounds. They examined children's and adults' processing of both reflexives and pronominal anaphora. In addition to finding that children are more likely than adults to be distracted by an inaccessible antecedent for a reflexive during processing, they also found a difference among adults between pronouns and reflexives in terms of the effect of inaccessible antecedents. Specifically, while reflexives were not susceptible to interference from inaccessible antecedents (unlike their findings with children), pronouns were to some extent, suggesting that even for adults the binding constraints do not eliminate feature matching noun phrases from consideration in the early stages of processing. The auditory stimuli in Clackson et al. (2011) manipulated whether the target anaphor was a pronoun or a reflexive and whether an inaccessible antecedent matched the anaphor in gender (Double match) or not (Single match), as in (6). In the second sentence of each condition, there is a matrix subject (*he/she*), and an embedded subject (*Mr. Jones*) which is the local subject of the clause containing the anaphor. For pronouns, the local subject is inaccessible according to Binding Theory whereas for reflexives the matrix subject is the inaccessible one. In both cases, the inaccessible antecedents are c-commanding subjects of the anaphor and thus are highly discourse prominent.

(6) Clackson et al. (2011) Experiment 2 auditory stimuli
  - Double match
    Peter was waiting outside the corner shop. He watched as Mr. Jones bought a huge box of popcorn for himself/him over the counter.
  - Single match
    Susan was waiting outside the corner shop. She watched as Mr. Jones bought a huge box of popcorn for himself/her over the counter.

While these sentences were presented auditorily, participants viewed a screen which displayed four pictures that represented the accessible referent, the inaccessible referent, the inanimate object referred to in the sentence (*huge box of popcorn*), and a distractor inanimate object. Of interest to our study are the results Clackson et al. (2011) obtained from adults. In the reflexive conditions, adults' eye-movements showed no effect of the inaccessible antecedent: looks to that referent were equally low whether or not the referent matched the gender of the reflexive anaphor (unlike with children). In the pronoun conditions, however, the gender of the inaccessible antecedent had an effect on eye-movement patterns. Adults, like children, showed fewer looks to the accessible antecedent in the double match condition than in the single match condition, suggesting that inaccessible antecedents interfere with pronoun reference resolution decisions when gender does not disambiguate. These results are consonant with Badecker and Straub's (2002) finding that inaccessible antecedents are entertained early in processing, but not with the *Simultaneous Hypothesis* (Chow et al., 2014) where both agreement and Binding Theory principles immediately and deterministically constrain the antecedent retrieval process for pronouns. The results are compatible with a weaker version of the latter view, where structural constraints interact with agreement constraints probabilistically in early processing (see Chow et al., 2014 p. 3, Parker and Phillips, 2017]. The difference between Chow et al. (2014) and Clackson et al. (2011) also raises the possibility that the methodology matters: perhaps the VWP could reveal an effect of inaccessible antecedents on pronouns that reading studies cannot, an issue we return to in section 5.

The findings of Clackson et al. (2011) (and Runner and Head, 2014) suggest that pronominal and reflexive anaphora are either sensitive to different constraints or to the same constraints but to different extents. Relevant here are VWP studies by Kaiser et al. (2009). They investigate anaphora in “picture noun phrases,” as in (7), where pronouns and reflexives are not entirely in complementary distribution as shown by the grammaticality of both options in (7).

(7) Mary_1_ saw a picture of herself_1_/her_1_.

In a series of VWP and offline studies, Kaiser et al. (2009) found that pronouns in picture noun phrases are less constrained by structural constraints than reflexives are, and are more sensitive to discourse-pragmatic factors, such as information source. They pitted structural constraints (a reflexive's need to find a subject antecedent and a pronoun's requirement not to) against the discourse/pragmatic factor of information source by manipulating verb type. With a verb such as *tell* the subject is the source of information and the object is the perceiver, whereas for *hear* the subject is the perceiver and the individual introduced in the PP *from* is the source.

(8)
  - Peter told Andrew about the picture of {him/himself} on the wall
  - Peter heard from Andrew about the picture of {him/himself} on the wall

Structural constraints predict that reflexives will uniformly seek the subject as antecedent and pronouns will seek the object as antecedent regardless of verb type. What Kaiser et al. (2009) found, however, was an asymmetry between pronouns and reflexives modulated by verb type along several measures. The pattern of looks to referents in the VWP very soon after participants heard the anaphor differed between pronouns and reflexives. Participants more consistently looked at the subject referent upon hearing reflexives regardless of verb type, whereas the looks after hearing the pronoun were more likely to the perceiver in both the *tell* and *hear* conditions. Thus the discourse-semantic manipulation affected pronoun processing more than reflexive processing. From these results, Kaiser et al. (2009) develop a form-specific multiple constraints model, whereby the processing of pronouns and reflexives differ in their sensitivity to the Binding Theory and where pronouns, in particular, are sensitive to discourse-pragmatic information at very early stages of processing. These findings could be taken to complement those found by Clackson et al. (2011), whereby the search for the referent of a pronoun may be less constrained by structural factors and more susceptible than reflexives to non-structural factors. Such a situation would be compatible with a number of views, including the one noted above in which structural and non-structural cues are probabilistically weighted in the search for a referent or antecedent. Further, in this view, this weighting is form-specific.

We think this conclusion is premature, however, since there is a feature of Clackson et al.'s (2011) stimuli that presents a possible confound when it comes to evaluating Principle B. In all of their stimuli, the anaphor (reflexive or pronoun) was housed inside a prepositional phrase (PP)^1^. Much as in picture noun phrases discussed above, pronouns and reflexives in PPs do not always neatly conform to the Binding Theory principles, as illustrated in (9). In (9a) and (9b), for instance, both a reflexive and a pronoun can corefer with the local matrix subject, even though this would violate Principle B in the case of pronouns. Similar examples that appear to violate Principle B, but are nonetheless well-formed, are given in (9c) and (9d).

(9)
  - Max_1_ saw a gun near himself_1_/him_1_.
  - Lucie_1_ counted five tourists in the room apart from herself_1_/her_1_.
  - Max_1_ put the gun near/under/on him_1_.
  - Max_1_ sat Lucie next to him_1_.

To explain these facts, Reinhart and Reuland (1993) argue that prepositions can form their own predicates independently of the verb, which has the effect of rendering the pronoun no longer a co-argument with the subject. The precise factors that determine whether a PP has this characteristic are complex (see Marantz, 1984 for discussion). What is relevant is that the stimuli in Clackson et al. (2011) involving pronouns consistently had the pronoun inside a PP. If pronouns in PPs are not subject to Principle B, then this presents a confound for Clackson et al. (2011). It could be that only pronouns in the canonical object position are subject to Principle B and would therefore show effects that would be expected if grammatical constraints had a very early effect on pronoun resolution.

It should also be pointed out that some of the PPs in Clackson et al.'s (2011) stimuli can also be best characterized as adjuncts (modifiers), as in (10a). That these are adjuncts can be confirmed by standard adjunct-argument diagnostics, including the fact that unlike arguments [e.g., *a large and colorful map* in (10a)], the PP (*for herself*) can be stranded by VP pro-form replacement [(10b) vs. (10c)].

(10)
  - Susan had drawn a large and colorful map for herself (Stimuli from Clackson et al., 2011).
  *Susan had done so a large and colorful map for herself.
  - Susan had done so for herself.

Adjuncts bear a different semantic and structural relationship within the clause than arguments do. Many theories of the distribution of pronouns and reflexives, including that of Reinhart and Reuland (1993), distinguish between Binding Conditions that hold of co-arguments and those that do not^2^.

For this reason, the present study employs stimuli where the pronoun and reflexive of interest are in an object argument position and are therefore unequivocally co-arguments with the local subject, as in (11).

(11)
  - Double match
    The young boy was waiting outside the corner shop. He watched as the old man who was wearing a hat bought himself/him a huge box of popcorn.
  - Single match
    The young girl was waiting outside the corner shop. She watched as the old man who was wearing a hat bought himself/her a huge box of popcorn.

In (11), the pronoun and reflexive anaphors are the first object (the indirect object) of the verb in a double object frame. These must be co-arguments of the subject (*the old man*) and therefore we expect these kinds of stimuli to be a more reliable test for the role of Principle B in early processing.

Our goal in this paper is to investigate the strength of Principle B effects on pronoun processing, in comparison to the strength of Principle A effects on reflexive processing, when pronouns and reflexives occur in an argument position, an environment properly subject to Binding Theory (Reinhart and Reuland, 1993). Contrary to Clackson et al. (2011) and Runner and Head (2014), we hypothesize that there are no form-specific differences between pronouns and reflexives and that the influence of Principle B on pronouns is as strong as the effect of Principle A on reflexives. As such, we predict that the amount of interference from a structurally inaccessible but gender-matched antecedent should be the same across pronouns and reflexives.

In what follows, we present three experiments. In Experiment 1 (forced-choice task), we tested whether the theoretical predictions about Binding Theory Principles A and B are borne out. In order to test the strength of Principles A and B in an on-line experiment, it was crucial that this off-line forced-choice task lent validity to the theoretical predictions of the Binding Principles. In Experiment 1, we found that adult native speakers of English make considered off-line judgments on both reflexives and pronouns in argument positions in accordance with Binding Principles A and B. The on-line profiles of pronoun and reflexive interpretation are tested in Experiment 2 (VWP eye-tracking), where we found significantly more looks to the accessible antecedent referent than the inaccessible antecedent referent for both pronouns and reflexives, and the amount of interference from the inaccessible antecedent for pronouns was no more than the amount of interference for reflexives. Experiment 3 (completion task) tested what types of arguments are expected when comprehenders encounter the critical verb. This was to ensure that the findings of Experiment 2 were not confounded by varying biases for a particular type of argument structure associated with the critical verbs. Experiment 3 showed that the critical verbs in our study were biased to be transitive, although they were instantiated as ditransitive in the test sentences in Experiment 2. However, the strength of this bias was the same across all tested conditions, and so it was not a confounding factor for the interpretation of the VWP results.

## 2. Experiment 1: Forced-Choice

We conducted a forced-choice task study to confirm that adult native speakers of English make considered off-line judgments for reflexives in accordance with Binding Principle A, and for pronouns in accordance with Binding Principle B.

### 2.1. Methods

#### 2.1.1. Participants

We recruited 19 native speakers of English from the university community. Each participant received a course credit upon completion of the experiment.

#### 2.1.2. Task, Design, and Materials

The test materials for Experiment 1 were adapted and modified from the auditory stimuli used in Clackson et al.'s (2011) Experiment 2, a VWP eye-tracking study. Participants were visually presented with a general context sentence (12a), followed by a pair of sentences [(12b) or (12c)]. The first of the pair introduces a human character [*the young boy* in (12b)] and further establishes a context. The second sentence of the pair, the target sentence, introduces another human character [*the old man* (12b)] as the subject of an embedded clause. This embedded clause is crucially ditransitive and contains the critical anaphor (pronoun or reflexive) as the indirect object, which is intended to refer to one of the two characters. The human characters in all stimuli were *the young boy, the young girl, the old man* or *the old woman*. The stimuli are manipulated so that the two characters both match in gender (Double match) or only one character matches in gender (Single match) with the critical pronoun or reflexive. In all cases, only one character is the grammatically licit antecedent of the critical anaphor. For example, in (12b), both *the young boy* and *the old man* match in gender with the reflexive *himself* or the pronoun *him*. However, the subject of the ditransitive clause, *the old man*, is the only grammatically licit antecedent for *himself*, and *the young boy* is the only grammatically licit antecedent for *him*. In (12c), only the subject of the ditransitive clause *the old man* matches in gender with *himself*, and it is also the grammatically licit antecedent for the reflexive. Further, only *the young girl* matches in gender with *her*, and it is also the grammatically licit antecedent for the pronoun. We will refer to the grammatically licit antecedent as the *accessible antecedent* and the grammatically illicit antecedent as the *inaccessible antecedent*. Participants were asked to choose which character in the sentence the indirect object corresponded with, by selecting one of the two possible antecedents. An example of the set of options given for the pronoun in (12b) is in (12d). The two answer options were only presented to participants 2 s after the sentences and the question were presented. This was done in order to prevent participants from rapidly running through the experiment, and to give them time to read the sentences prior to seeing the answer options.

(12)
  - Context
    It was the first day of Summer vacation.
  - Double match
    The young boy was spending a day at the beach. He was amazed to see that the old man who was carrying a bucket built himself/him a magnificent sand castle.
  - Single match
    The young girl was spending a day at the beach. She was amazed to see that the old man who was carrying a bucket built himself/her a magnificent sand castle.
  - Question
    Who does “him” refer to?
    The old man   The young boy

Twenty-four test items, as in (12), were created, testing two factors with two levels each, Match (Double match vs. Single match) and Anaphor (Reflexive vs. Pronoun), yielding four conditions. The 24 test items were distributed over four lists in a Latin-square design so that no participant saw any one item in more than one condition.

In addition to the 24 test items, 24 filler items were created, as in (13), and added to the four lists. These fillers were adapted and modified from the materials used in Grant et al.'s (2020) Experiment 2, so that they contained pronouns which are unambiguous with only one possible antecedent mentioned in the sentence. For instance, in (13), the pronoun *she* can only refer to *the young princess*. The other nominal expression, *the revered king*, is structurally accessible but constitutes a gender mismatch. The purpose of this was to ensure that the participants were executing the task appropriately.

(13)
  - Context
    Picking a new leader is serious work.
  - Filler item
    The people were very impressed. The young princess showed the revered king that she would be a fine leader of the Tharassian empire.
  - Question
    Who does “she” refer to?
    The young princess   The revered king

#### 2.1.3. Procedure

Participants were directed to the online experiment platform PennController for IBEX (Zehr and Schwartz, 2018). They received instructions and completed two practice trials before proceeding with the experimental trials. After completing the experiment, they filled out a brief demographic survey.

### 2.2. Results

**Figure 1:**
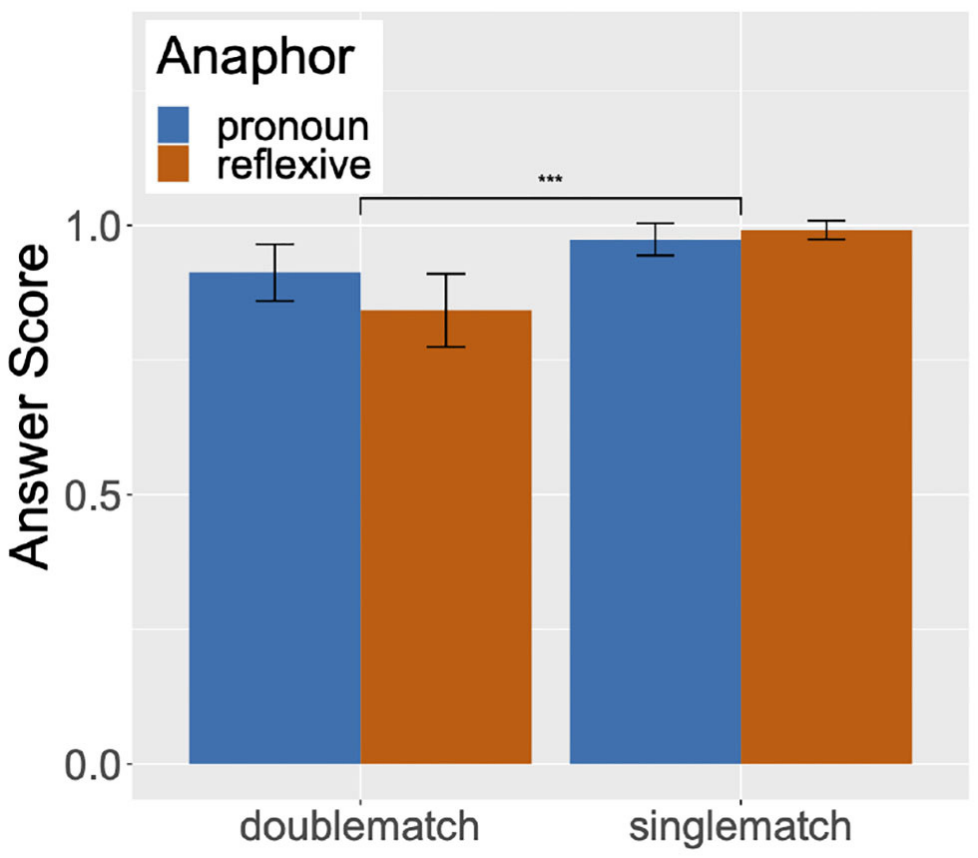
Mean answer scores corresponding to structurally accessible antecedent selection in single/double match conditions, Experiment 1.

For each trial, the selection of a structurally accessible antecedent was scored as 1, and the selection of a structurally inaccessible antecedent was scored as 0. The graph in Figure 1 summarizes the mean answer score by condition. Numerically, the double match condition has a lower mean answer score than the single match condition, with the reflexives in the double match condition exhibiting the lowest mean.

We analyzed the answer scores by means of a generalized mixed-effects model (logistic/binomial regression model) in R (R Development Core Team, 2012). The lme4 package was used to fit the model (Bates et al., 2012), and the lmerTest package was used to obtain *p*-values (Kuznetsova et al., 2017). In analyzing the data, we fit a random-effects structure with random intercepts for participants and items. Random slopes were not included as their inclusion did not lead to a successful model convergence (Barr et al., 2013).^3^

The model was fit to the answer scores with fixed factors of Match (Single match vs. Double match) and Anaphor (Reflexive vs. Pronoun). These predictors were sum coded, with one of the levels coded as 1 (Pronoun in Anaphor and Double match in Match), and the other as –1 (Reflexive in Anaphor and Single match in Match). We chose to use sum-to-zero coding schema to center each contrast (Sonderegger et al., 2018). We found a significant effect of Match (Estimate = –1.12, *SE* = 0.28, *z* = –3.94, *p* < 0.001), but no interaction of Match and Anaphor^4^. Upon planned comparisons between the answer scores of the double match and the single match conditions for pronouns and reflexives, a significant difference was found for both comparisons, with lower answer scores in the double match condition than the single match condition (pronoun: Estimate = –0.75, *SE* = 0.39, *z* = –1.96, *p* < 0.05; reflexive: Estimate = –1.79, *SE* = 0.55, *z* = –3.24, *p* < 0.01). The findings from the planned comparisons and the finding of no interaction of Match and Anaphor taken together indicate that the mean answer score in the double match condition was significantly lower than the score in the single match condition for both pronouns and reflexives, and to the same extent for both anaphor types.

The mean answer score for the fillers was 0.94, indicating that the correct antecedent was selected for unambiguous pronouns in most cases. This suggests that participants were executing the task appropriately, and lends some support to the validity of the results for the test items.

### 2.3. Discussion

The results of Experiment 1 show that adult native speakers of English generally make considered off-line judgments on reflexives in accordance with Binding Principle A and on pronouns in accordance with Binding Principle B. However, for both pronouns and reflexives, native speakers sometimes make mistakes by selecting a structurally inaccessible antecedent when it matches in gender with the critical anaphor. This can be seen by the fact that the answer scores for the double match condition for both pronouns and reflexives are slightly lower than the single match condition, and this difference was statistically significant. Importantly, the extent to which an inaccessible antecedent was selected over the accessible antecedent was the same for both pronouns and reflexives, as supported by the absence of interaction of Match and Anaphor. In fact, the mean answer score for the reflexives in the double match condition was numerically lower than that for pronouns in the same condition. Hence, we found no evidence that the effect of Principle B on pronouns is weaker than the effect of Principle A on reflexives in off-line judgments.

## 3. Experiment 2: Visual World Paradigm Eye-Tracking

The purpose of our VWP eye-tracking study was to test whether the extent to which pronouns are constrained by Binding Principle B is equal to the extent to which reflexives are constrained by Binding Principle A in the early stages of anaphoric processing. Using eye-movement data, we examined the level of competition between structurally accessible and inaccessible antecedents, and compared the amount of interference from structurally inaccessible antecedents during pronoun and reflexive interpretation. Unlike Clackson et al. (2011) we employed stimuli where the relevant anaphor was a nominal argument rather than a constituent within a PP, as these are known to exhibit different binding properties (Reinhart and Reuland, 1993), as discussed in the section 1.

### 3.1. Methods

#### 3.1.1. Participants

We tested 28 native speakers of English who had normal or corrected vision, recruited from the university community. None of them had participated in Experiment 1^5^. Each participant was paid CAD $10 or given course credit for taking part in the experiment.

#### 3.1.2. Task, Design, and Materials

The task, design and materials used for Experiment 2 were adapted and modified from Clackson et al.'s (2011) Experiment 2. Participants were presented with a combination of visual and auditory stimuli. The auditory stimuli came from Experiment 1. In each trial, while viewing a display on a computer screen, participants heard two sentences introducing two human characters [(14a) or (14b)]. The second sentence contains the critical anaphor (pronoun or reflexive) in the embedded ditransitive clause as the indirect object. The subject of the ditransitive clause is modified by a relative clause (*who was carrying a bucket*) that includes an inanimate object (*a bucket*). An inanimate object was introduced here in order to direct participants' gaze away from the images of the two human characters to the image of the inanimate object before the onset of the target anaphor. The human characters in all auditory stimuli were *the young boy, the young girl, the old man*, or *the old woman*. The experiment consisted of 24 test items and followed a 2 × 2 factorial design crossing Match (Double match vs. Single match) and Anaphor (Reflexive vs. Pronoun). The characters in the target sentences either both matched the gender of the anaphor [Double match in (14a)], or only one character matched the gender of the anaphor [Single match in (14b)]. The 24 test items were distributed over four lists in a Latin-square design.

(14)
  - Double match
    The young boy was spending a day at the beach. He was amazed to see that the old man who was carrying a bucket built himself/him a magnificent sand castle.
  - Single match
    The young girl was spending a day at the beach. She was amazed to see that the old man who was carrying a bucket built himself/her a magnificent sand castle.
  - Forced-choice comprehension question
    Did the old man build a magnificent sand castle?

In addition to 24 test items, 24 filler items, as in (15), were created and added to the four lists. Fillers had a similar format as the test items: a pair of sentences introducing two human characters, a ditransitive clause and an inanimate object in a relative clause. However, unlike the test items, the fillers did not have any pronouns or reflexives and contained only referring expressions. All filler trials involved the same human characters as the test trials.

(15)
  - Filler item
    It was late Friday afternoon. At the theater, the old man who was holding a glass of coke bought the young boy a huge box of snacks.
  - Forced-choice comprehension question
    Was the old man holding a glass of coke?

**Figure 2:**
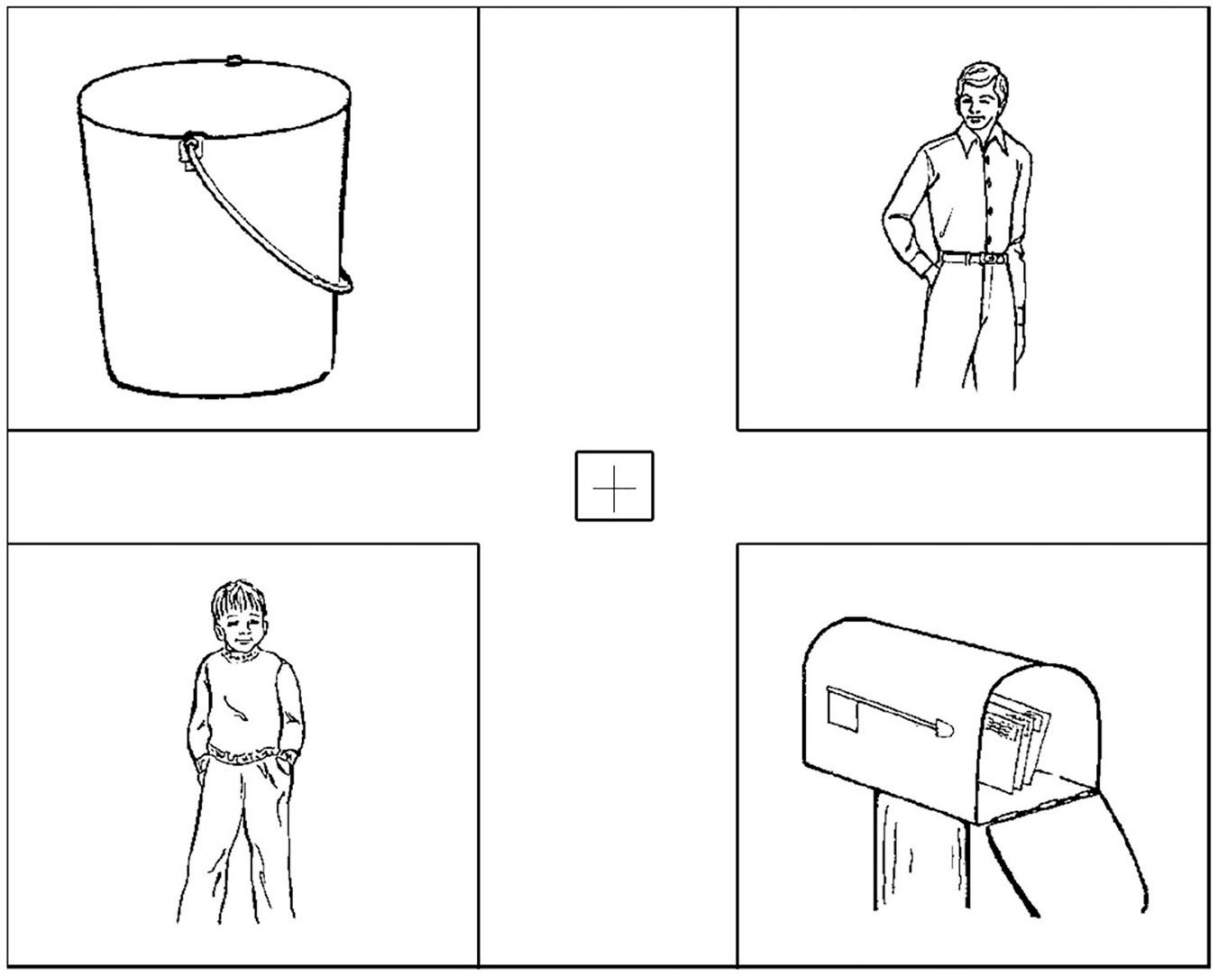
Display of the trial corresponding to (14a), Experiment 2.

The display contained four images: images of the two human characters mentioned in the sentences, an image of the inanimate object mentioned in the relative clause in the second sentence, and an image of an inanimate distractor object not mentioned in the sentences. An example display for a double match condition is given in Figure 2. The four images were placed in the four corners of the computer screen, and a cross was placed in the middle of the screen. Each image in Figure 2 is surrounded by a rectangle to show how its interest region on the screen was determined. Note that these rectangular markings were not visible to the participants. The order of the human character images and the inanimate object images was counterbalanced across all trials. The order of the images from top-left to bottom-right was randomly determined upon beginning the experiment for each trial. Each possible ordering of the images had an equal probability of occurring.

After hearing the two sentences, participants were aurally given a forced-choice yes-no comprehension question in a subsequent display (14c). The participants' task was to answer the question by clicking on the appropriate answer box. The comprehension questions tested participants' understanding of the sentences, but not their interpretation of the critical word, to ensure that they were paying attention to the task. Half of the test and filler trials had *yes* as a correct answer and the other half had *no* as the correct answer.

Sound files of the sentence pairs in all test and filler trials were recorded by a female native speaker of English, and sound files of all comprehension questions were recorded by a male native speaker of English. All images we used were black and white pictures downloaded from the International Picture Naming Project website (https://crl.ucsd.edu/experiments/ipnp/1stimuli.html).

#### 3.1.3. Procedure

The images were presented on a PC using Experiment Builder (SR Research LTD, 2014). The participants' responses to forced-choice questions were also recorded using Experiment Builder. They heard the pre-recorded sound files associated with display images through headphones. Eye-tracking measures were taken using a desktop Eyelink 1000 eye-tracker (SR Research LTD, 2010), sampling at 1000 Hz. Participants were tested individually in a private testing booth in a lab.

Upon arriving at the lab, participants were briefed on the nature of their task, signed consent forms, and were introduced to the eye-tracking equipment by completing a 9-point calibration routine. After calibration, participants were introduced to the images of each character in the experiment individually, alongside an audio description of that character (e.g., “This is the old man.”). Following that, they saw two practice trials using images and sentences not repeated during the experiment. These trials were designed to familiarize participants with the audio-visual combination, and to get them accustomed to the self-pacing of the experiment by way of their responses to the comprehension questions. Each participant saw 24 test trials (six per condition) and 24 filler trials in a uniquely generated random order. At the beginning of each trial, a screen with a fixation cross in the center was displayed to serve as a cue to draw the participants' gaze back to the center of the screen. Each trial began once participants fixated on the cross for at least 100 ms. Participants used a chin rest throughout the duration of the experiment.

Once the entire experiment was completed, participants were given a written debriefing form, as well as an informal verbal debriefing with the experimenter.

#### 3.1.4. Predictions

We tracked eye-movements of the participants from the onset of the target anaphor. We will consider two hypotheses and the pattern of looks each of them predicts: (i) that the Principle B effect on pronouns is as strong as the Principle A effect on reflexives, and (ii) that the Principle B effect on pronouns is weaker than the Principle A effect on reflexives.

If the strength of the Principle B effect on pronouns is the same as the Principle A effect on reflexives, then anaphoric resolution for pronouns should be no more susceptible to interference from structurally inaccessible yet feature-matching antecedents than anaphoric resolution for reflexives. In the single match condition, only the accessible antecedent matches in gender with the pronoun or the reflexive, and so, regardless of the strength of the Binding Condition effects, we expect minimal interference from the inaccessible antecedent for both pronouns and reflexives. In the double match condition, Binding Conditions A and B should rule out the gender-matched inaccessible antecedent as a possible antecedent and so it should not interfere with the anaphoric resolution of either pronouns or reflexives. Therefore, for both pronouns and reflexives, the amount of looks to the inaccessible antecedent image in the double match condition should not be higher than in the single match condition.

If the Principle B effect on pronouns is weaker than the Principle A effect on reflexives, then pronouns should be more susceptible to interference from feature matching but structurally inaccessible antecedents than reflexives. Thus, while we do not expect to see different patterns of looks between the two anaphors in the single match condition, different patterns should emerge in the double match condition. In the single match condition, as only the accessible antecedent matches in gender with the target anaphor, we again expect minimal interference from the inaccessible antecedent for pronouns as well as for reflexives, regardless of the strength of the Binding Condition effects. However, in the double match condition, if pronouns are more susceptible to interference than reflexives, then for pronouns but not for reflexives, we expect to see a higher amount of looks to the inaccessible antecedent image than in the single match condition.

### 3.2. Results

#### 3.2.1. Comprehension Question Response Accuracy

The mean proportions of correct responses for the comprehension questions are reported in Table 1. Although the comprehension questions did not themselves test for the interpretation of the critical anaphors (they merely tested participants' attention to the sentence content), the high accuracy rates across the board (93% or higher) show no impact of the manipulated factors on comprehension generally and that participants were paying attention to the task. No participant fell below 79% accuracy.

**Table 1:** Proportions of correct responses (SE), Experiment 2.

	**Double match**	**Single match**
Pronoun	0.93 (0.001)	0.93 (0.001)
Reflexive	0.93 (0.001)	0.95 (0.001)

#### 3.2.2. Overall Pattern of Looks to Accessible/Inaccessible Antecedents

The way the interest region around each image was determined on the screen is marked with a rectangular box in Figure 2. A 500 × 450 pixel interest region was centered around each of the human and object images. Each interest region extended to the corner of the monitor in case of eye-drift throughout the experiment or any potential issues with accurately capturing eye positions near the edge of the monitor. The crosshair in the center of the screen was contained within a 50 × 50 pixel interest region. The interest region covering the crosshair was used to initiate each trial, requiring each participant's eyes to be at the center of the screen prior to the onset of the target sentence audio. No other interest regions were defined. Using Eyelink Dataviewer (SR Research LTD, 2010), we produced a binned sample report in which counts of looks to the interest regions for the accessible antecedent, inaccessible antecedent, target object, and distractor object were recorded at every 20 ms. The count of all on-screen looks that did not fall on any of the four interest regions was also recorded. We will refer to the areas on the screen that are not part of the four interest regions as the null region. 6% of all looks recorded were to the null region. A proportion of looks to an image for each 20 ms time period was calculated by dividing the sum of looks recorded in the interest region of that image by the total sum of looks recorded in all four interest regions and the null region. Missing data accounted for 17% of the total dataset from the onset of the anaphor to 2,000 ms after the onset. Off-screen looks and blinks were treated as missing data. Off-screen looks accounted for 2% of the total dataset, and blinks accounted for 9% of the total dataset. For missing data, the counts of looks in all four interest regions and the null region were recorded as zero.

**Figure 3:**
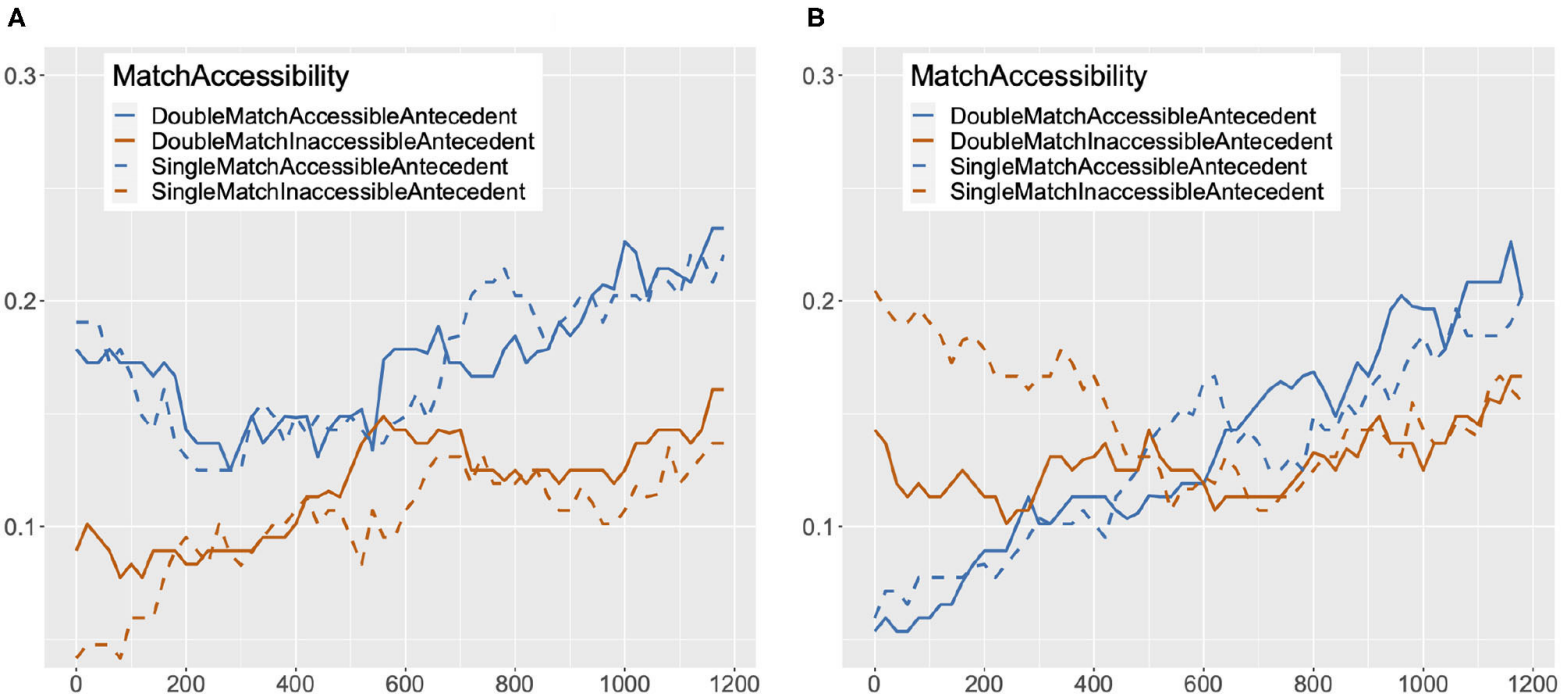
Proportion of looks to accessible/inaccessible antecedent image in single/double match condition at every 20 ms from the onset of the target anaphor to 1,200 ms after the onset, Experiment 2. **(A)** Proportion of Looks: Reflexive. **(B)** Proportion of Looks: Pronoun.

The proportions of looks to accessible and inaccessible antecedents in double match and single match conditions are plotted at every 20 ms from the onset of the target reflexive to 1,200 ms after the onset in Figure 3A. Proportions of looks for the target pronoun are plotted in Figure 3B. The data in these figures are structured and summarized in a similar way as in Clackson et al.'s (2011), Figures 1, 4 in Experiment 2.

**Figure 4:**
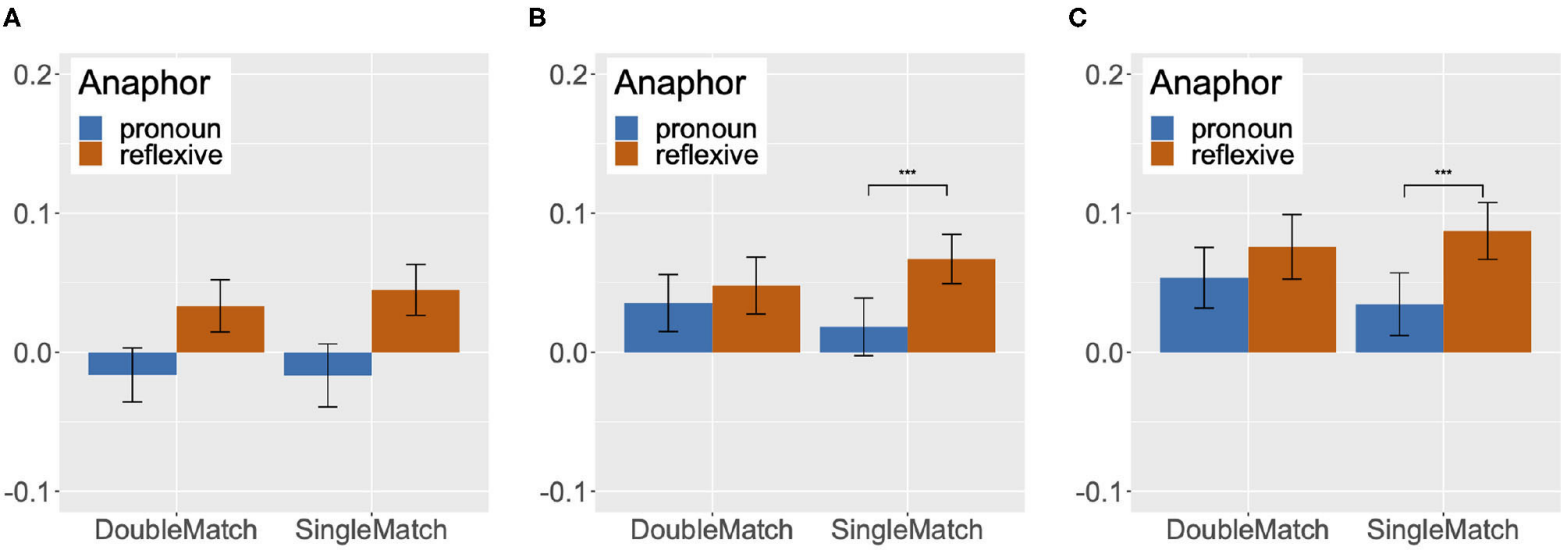
Difference between looks to accessible and inaccessible antecedent images in single/double match condition, grouped into 300 ms time periods from 300 ms after the onset of the target anaphor to 1,200 ms after the onset, Experiment 2. **(A)** BT score, 301-600. **(B)** BT score, 601-900. **(C)** BT score, 901-1200.

For reflexives, at the onset there are already more looks to the accessible antecedent image than the inaccessible antecedent image in both the single match and the double match condition. This early pattern of looks should be attributed to the fact that the most recently mentioned person [e.g., *the old man* in (14)] corresponds to the accessible antecedent for reflexives. The proportion of looks to the accessible antecedent image starts to increase at around 550 ms after the onset in the double match condition, and slightly after that in the single match condition. The proportion of looks to the inaccessible antecedent image remains lower than the proportion of looks to the accessible antecedent image throughout, with a slight rise in looks to the gender-mismatched inaccessible antecedent image starting at around 100 ms after the onset, and a slight rise in looks to the gender-matched inaccessible antecedent image at around 450 ms after the onset. A clear differentiation between accessible and inaccessible antecedents can be seen in both the double match and the single match condition from around 700 ms, with a higher proportion of looks to the accessible than the inaccessible antecedent.

For pronouns, there are more looks to the inaccessible antecedent image than the accessible antecedent image at the onset. This can be attributed to the fact that for pronouns, the most recently mentioned person [e.g., *the old man* in (14)] corresponds to the inaccessible antecedent. However, the proportion of looks to the accessible antecedent image starts to gradually increase at the onset, surpassing the proportion of looks to the inaccessible antecedent image at around 500 ms in the single match condition, and at around 600 ms in the double match condition. On the other hand, looks to the inaccessible antecedent image do not increase in frequency: the proportion of looks to the inaccessible antecedent image starts to decrease at the onset, leveling out early for the gender-matched inaccessible antecedent, and at around 500 ms for the gender-mismatched inaccessible antecedent. In comparison to reflexives, however, a clear differentiation between accessible and inaccessible antecedents is seen later and to a lesser degree, after 900 ms^6^.

To statistically analyze the eye-movement data, generalized linear mixed-effects models (logistic/binomial regression models) were fit to the data using the glmer function of the lme4 package (Bates et al., 2012) in R (R Development Core Team, 2012), and the lmerTest package was used to obtain *p*-values (Kuznetsova et al., 2017). As the dependent measure of the eye-movement data, for each 20 ms time period, we used a binary value (0 or 1) indicating whether or not a participant looked at the image corresponding to a potential antecedent: a score of 1 was assigned if a participant fixated on the potential antecedent image, and a score of 0 was assigned otherwise.

In our analyses of all the data reported in Experiment 2, we first attempted to fit a maximal random-effects structure with random intercepts and random slopes for participants and items (Barr et al., 2013). If that model did not converge, we fit a model just like the maximal model, but with the random correlation parameter for the interaction term removed for both participants and items.

Below, we first present the results of fitting separate mixed models to the fixation data for reflexives and pronouns between 301 and 1,200 ms from the onset of the target anaphor. Separate mixed models were also fit to the reflexive and pronoun data in Clackson et al. (2011). We then present the results of an analysis that directly compares the data for reflexives and pronouns. We chose to analyze the fixation data starting from 301 ms from the onset because previous studies have found that fixations to targets usually diverge from competitors only after 300 ms after the onset of the relevant word (Allopenna et al., 1998; Dahan and Tanenhaus, 2004; Runner et al., 2006). We also chose to analyze the data up to 1,200 ms following the onset as this time point on average coincided with the end of the target sentence.

As in Clackson et al. (2011), the separate models for reflexives and pronouns were fit to the data with fixed effects of Bin Index (for each 20 ms time period), Antecedent (Accessible vs. Inaccessible) and Match (Single match vs. Double match). Unlike in Clackson et al. (2011), however, Bin Index was assumed to be linear, as adding a second order polynomial Bin Index did not improve the fit of the model. Antecedent and Match were sum coded, with one of the levels coded as 1 (Accessible in Antecedent and Double match in Match), and the other as –1 (Inaccessible in Antecedent and Single match in Match). Bin Index was grand-mean centered. Moreover, in the analysis that directly compared the data for reflexives and pronouns, a fixed effect of Anaphor (Pronoun vs. Reflexive) was added to the model, with the sum coding of 1 assigned to Pronoun and –1 to Reflexive.

For both pronouns and reflexives in the single match condition, we expect to see significantly more looks to the accessible antecedent image than to the inaccessible antecedent image, regardless of the strength of the Binding Conditions, as only the accessible antecedent matches in gender with the target anaphor. For reflexives in the double match condition, given the findings in the previous literature (Nicol and Swinney, 1989; Harris et al., 2000; Sturt, 2003; Xiang et al., 2009; Clackson et al., 2011) that the anaphoric resolution of reflexives is sensitive to Binding Theory at the early stages of processing, we expect to see significantly more looks to the accessible antecedent image than to the inaccessible antecedent image. As for pronouns in the double match condition, if the Principle B effect on pronouns is as strong as the Principle A effect on reflexives, we should also see significantly more looks to the accessible antecedent image than to the inaccessible antecedent image. But if the Principle B effect on pronouns is weaker than the Principle A effect on reflexives, then the amount of looks to the inaccessible antecedent image should be different between the single match and the double match conditions, with increased looks to the gender-matched inaccessible antecedent in the double match condition. This should result in a smaller difference between the amount of looks to the accessible antecedent and to the inaccessible antecedent in the double match condition.

In our analysis of the reflexives, we found a significant effect of Antecedent, as shown in Table 2^7^. Planned comparisons between the looks to the accessible antecedent image and the inaccessible antecedent image in the single match and the double match conditions showed that in both conditions, there were more looks to the accessible antecedent image than to the inaccessible antecedent image (single match: Estimate = 0.31, *SE* = 0.03, *z* = 12.32, *p* < 0.001; double match: Estimate = 0.23, *SE* = 0.02, *z* = 9.47, *p* < 0.001).

**Table 2:** Fixed effects from best fitting mixed-effects logistic regression model fit to data for reflexives, Experiment 2.

**Fixed effects**	**Estimate**	**SE**	**z**	**p**
(Intercept)	−2.37	0.31	−7.71	<0.001^***^
BinIndex	0.02	0.01	2.62	0.009^**^
Antecedent(Accessible)	0.44	0.17	2.53	0.01^*^
Match(DoubleMatch)	0.14	0.14	1.05	0.30
Ant(Access)×Match(Double)	−0.01	0.02	−0.54	0.59
Formula in R: Fixation ~ BinIndex + Antecedent ^*^ Match + (1 + BinIndex + Antecedent + Match | Participant) + (1 + BinIndex + Antecedent + Match | Item)				

Significance levels:

*** *p < 0.001*,

** *p < 0.01*,

* *p < 0.05*,

*^+^p < 0.1*.

In our analysis of the pronouns, we found an interaction of Antecedent and Match, as shown in Table 3^8^. However, upon planned comparisons between the looks to the accessible antecedent image and the inaccessible antecedent image in the single match and the double match conditions, a significant difference was found in both conditions, with more looks to the accessible antecedent image than to the inaccessible antecedent image (single match: Estimate = 0.06, *SE* = 0.02, *z* = 2.28, *p* = 0.02; double match: Estimate = 0.11, *SE* = 0.02, *z* = 4.53, *p* < 0.001). Looking at the estimates and *z* values, the interaction seems to be due to the smaller difference between the amount of looks to the accessible antecedent and the inaccessible antecedent in the single match condition. This is consistent with the pattern of proportion of looks for pronouns visualized in Figure 3B. There, it can be seen that looks to the accessible antecedent in the single match condition are reduced in comparison to the double match condition soon after 600 ms following the onset of the target pronoun. The amount of looks to the inaccessible antecedent in the single match condition stays similar to the amount in the double match condition during this time period.

**Table 3:** Fixed effects from best fitting mixed-effects logistic regression model fit to data for pronouns, Experiment 2.

**Fixed effects**	**Estimate**	**SE**	**z**	**p**
(Intercept)	−2.34	0.23	−10.35	<0.001^***^
BinIndex	0.02	0.007	2.46	0.01^*^
Antecedent(Accessible)	0.14	0.18	0.75	0.45
Match(DoubleMatch)	0.02	0.17	0.13	0.90
Ant(Access)×Match(Double)	0.06	0.02	2.79	0.005^**^
Formula in R: Fixation ~ BinIndex + Antecedent ^*^ Match + (1 + BinIndex + Antecedent + Match | Participant) + (1 + BinIndex + Antecedent + Match | Item)				

Significance levels:

*** *p < 0.001*,

** *p < 0.01*,

* *p < 0.05*,

*^+^p < 0.1*.

In the analysis that directly compared the data for reflexives and pronouns, we found a significant effect of Antecedent, a two-way interaction of Antecedent and Anaphor, and a three-way interaction of Antecedent, Match and Anaphor, as shown in Table 4^9^. The presence of an interaction of Antecedent and Match in the pronoun data and the absence of it in the reflexive data, as we have seen in the separate analyses for the two anaphor types, resulted in a three-way interaction of Antecedent, Match and Anaphor. Thus, the results show that there is a higher amount of looks to the accessible antecedent than the inaccessible antecedent overall, in particular for reflexives, but this is due to the reduced looks to the accessible antecedent for the pronouns in the single match condition in comparison to the pronouns in the double match condition and to the reflexives in the single/double match condition after 600 ms. The amount of looks to the inaccessible antecedent for pronouns in the single match condition is similar to the amount in other conditions during this time period.

**Table 4:** Fixed effects from best fitting mixed-effects logistic regression model fit to data for reflexives and pronouns, Experiment 2.

**Fixed effects**	**Estimate**	**SE**	**z**	**p**
(Intercept)	−2.08	0.20	−10.53	<0.001^***^
BinIndex	0.02	0.005	3.25	0.001^**^
Antecedent(Accessible)	0.24	0.11	2.25	0.03^*^
Match(DoubleMatch)	0.05	0.09	0.57	0.57
Anaphor(Pronoun)	0.05	0.09	0.62	0.54
Ant(Access)×Match(Double)	0.01	0.01	0.67	0.50
Ant(Access)×Anaphor(Pron)	−0.13	0.01	−9.53	<0.001^***^
Match(Double)×Anaphor(Pron)	−0.02	0.01	−1.43	0.15
Ant(Access)×Match(Double)×Anaphor(Pron)	0.05	0.01	4.33	<0.001^***^
Formula in R: Fixation ~ BinIndex + Antecedent ^*^ Match ^*^ Anaphor + (1 + BinIndex + Antecedent + Match + Anaphor | Participant) + (1 + BinIndex + Antecedent + Match + Anaphor | Item)				

Significance levels:

*** *p < 0.001*,

** *p < 0.01*,

* *p < 0.05*,

*^+^p < 0.1*.

#### 3.2.3. Binding Theory Score

We obtained a measure corresponding to the strength of Binding Conditions for reflexives and pronouns by aggregating the fixation scores of potential antecedents for each 20 ms time period by participant, and subtracting the fixation score of inaccessible antecedents from the fixation score of accessible antecedents. We will refer to this measure as the Binding Theory score. We can assume that the bigger the score, the greater the strength of Binding Theory principles.

We analyzed the Binding Theory score as a function of Anaphor (Reflexive vs. Pronoun) and Match (Single match vs. Double match) by means of a mixed-effects model in R (R Development Core Team, 2012). This analysis is another way of directly comparing the strength of the Binding Condition effects on pronouns and reflexives, in addition to the mixed-effects analysis that directly compared the fixation data for pronouns and reflexives in Table 4. The lme4 package was used to fit the model (Bates et al., 2012), and the lmerTest package was used to obtain *p*-values (Kuznetsova et al., 2017). All predictor variables were sum coded, with one of the levels coded as 1 (Pronoun in Anaphor and Double match in Match), and the other as –1 (Reflexive in Anaphor and Single match in Match). In order to identify any time course effects, we divided the 301–1,200 ms window into three 300 ms time periods (301–600, 601–900, and 901–1,200 ms), and performed a separate analysis for each time period. We chose to divide the 301–1,200 ms window into three equal time periods in order to facilitate discussion of any differences in the strength of Binding Conditions, if detected, in the early, middle and later part of the analyzed time window. A similar method was used in Kaiser et al. (2009), where their 2,000 ms time window was divided into five 400 ms time periods for analysis.

If the Principle B effect on pronouns is as strong as the Principle A effect on reflexives, there should be no difference between the Binding Theory scores of pronouns and reflexives in the double match condition. However, if the Principle B effect on pronouns is weaker than the Principle A effect on reflexives, reflexives should show higher Binding Theory scores than pronouns in the double match condition. In the single match condition, we do not expect to find any difference between the Binding Theory scores of the two types of anaphors, regardless of the strength of the Binding Conditions, as there is only one gender-matched antecedent which is also the accessible antecedent for both pronouns and reflexives.

The graphs in Figure 4 summarize mean Binding Theory scores by condition in the three time periods. In the 301–600 ms time period, we found no significant fixed effect or interaction, despite the fact that the mean Binding Theory score of reflexives was numerically higher than that of pronouns in both the double match and the single match condition. As noted by a reviewer, this numerical trend can be explained by the effect of recency: the most recently mentioned person [e.g., *the old man* in (14)] corresponds to the accessible antecedent in the reflexive condition and to the inaccessible antecedent in the pronoun condition.

In the 601–900 ms time period, we found an interaction of Match and Anaphor (Estimate = 0.009, *SE* = 0.003, *t* = 2.98, *p* < 0.01). Upon planned comparisons between the pronouns and the reflexives in the single match and the double match condition, we found a significant difference in the single match condition (Estimate = –0.02, *SE* = 0.01, *t* = –3.89, *p* < 0.001), but not in the double match condition (Estimate = –0.01, *SE* = 0.01, *t* = –1.05, *p* > 0.05). The interaction was thus due to the fact that the Binding Theory scores for reflexives and pronouns were similar in the double match condition, but reflexives showed a much higher Binding Theory score than pronouns in the single match condition.

An interaction of Match and Anaphor was also found in the 901–1,200 ms time period (Estimate = 0.007, *SE* = 0.003, *t* = 2.41, *p* < 0.05). Just as in the 601–900 ms time period, planned comparisons between the pronouns and the reflexives revealed a significant difference in the single match condition (Estimate = –0.03, *SE* = 0.01, *t* = –4.07, *p* < 0.001), but not in the double match condition (Estimate = –0.01, *SE* = 0.01, *t* = –1.71, *p* > 0.05). So, the interaction of Match and Anaphor was the result of the higher Binding Theory score of reflexives in the single match condition.

### 3.3. Discussion

In our analysis of the overall pattern of looks, we found significantly more looks to the accessible antecedent image than the inaccessible antecedent image in both the double match and the single match conditions for reflexives. For pronouns, although there were more looks to the accessible antecedent image than to the inaccessible antecedent image in both the single and the double match conditions, the difference between the amount of looks to the two types of antecedent images was more robust in the double match condition than in the single match condition. This was due to a reduction in looks to the accessible antecedent image in the single match condition. The finding that pronouns and reflexives pattern alike in the double match condition is consistent with the hypothesis that the Principle B effect on pronouns is as strong as the Principle A effect on reflexives. The finding that pronouns in the single match condition exhibited reduced looks to the accessible antecedent image is unexpected, however. As only the accessible antecedent matched in gender with the target pronoun, looks to the accessible antecedent image should not have been affected, regardless of the strength of the Binding Condition effects.

In our analysis of the Binding Theory scores, in the 301–600 ms time period, we saw a numerical trend that reflexives had higher Binding Theory scores than pronouns in both the double match and the single match conditions, although this trend was not statistically significant. As noted earlier, these apparently higher Binding Theory scores for reflexives in this early time period can be explained by the fact that in all conditions, there were high proportions of fixations to the image of the subject of the clause [e.g., *the old man* in (14)], which corresponds to the accessible antecedent in the reflexive condition, at the onset of the pronoun or the reflexive, as this was the last mentioned person in the sentence. This point will be discussed in more detail shortly. In both the 601–900 ms and the 901–1,200 ms time periods, pronouns showed Binding Theory scores that were just as high as reflexives in the double match condition, but showed lower Binding Theory scores than reflexives in the single match condition. Thus, the difference between the amount of looks to the accessible antecedent image and the inaccessible antecedent image was similar across pronouns and reflexives in the double match condition, with more looks to the accessible antecedent than to the inaccessible antecedent. But in the single match condition, pronouns showed a smaller difference between the amount of looks to the accessible antecedent image and to the inaccessible antecedent image in comparison to reflexives. The finding that pronouns and reflexives have similar Binding Theory scores in all time periods in the double match condition is consistent with the hypothesis that the Principle B effect on pronouns is as strong as the Principle A effect on reflexives. But the finding that pronouns exhibited lower Binding Theory scores than reflexives in the single match condition is unexpected. This was again due to a reduction in looks to the accessible antecedent image for pronouns in the single match condition.

The fact that reflexives had higher Binding Theory scores than pronouns in the early time period (301–600 ms), although not statistically significant, might be an indication that the Principle B effect on pronouns kicks in slightly later than the Principle A effect on reflexives. We also see potential support for this view in the graphs in Figure 3: at the onset of the reflexives, there are already more looks to the accessible antecedent image than the inaccessible antecedent image, but for pronouns, it takes some time for the proportion of looks to the accessible antecedent image to surpass the proportion of looks to the inaccessible antecedent image. However, this apparent difference in the timing of the Binding Condition effects may be partially due to a delayed response to the co-argument subject of the clause, the most recently mentioned person in the sentence, which corresponds to the accessible antecedent for reflexives and the inaccessible antecedent for pronouns. For example, *the old man* is the co-argument subject of the reflexive or pronoun in (14a) and (14b). It may be that some participants were still sustaining their attention to the referent of *the old man*, fixating on that image even after the onset of the target anaphor.

**Figure 5:**
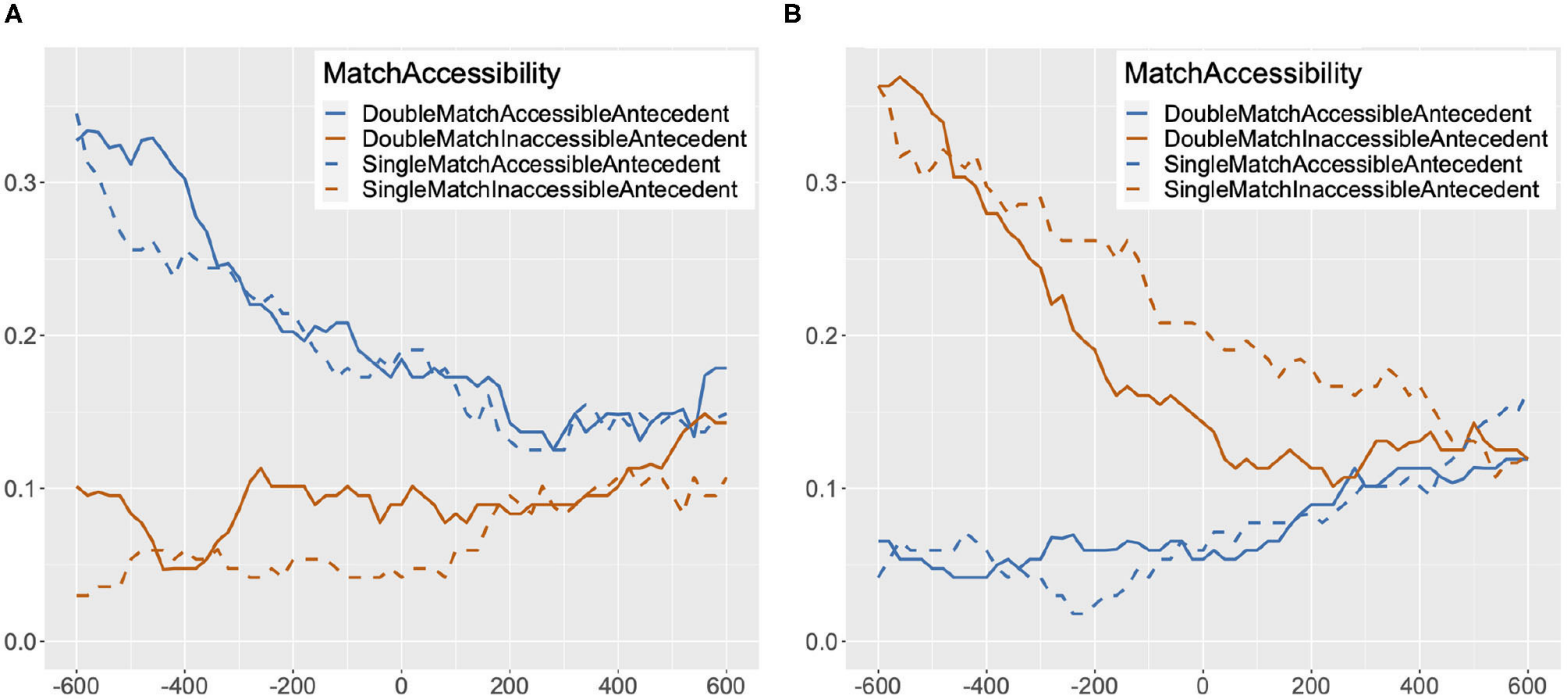
Proportion of looks to accessible/inaccessible antecedent image in double/single match condition at every 20 ms from 600 ms before the onset of the target anaphor to 600 ms after the onset, Experiment 2. **(A)** Proportion of Looks: Reflexive. **(B)** Proportion of Looks: Pronoun.

The materials were designed so that an inanimate object [e.g., *a bucket* in (14)] was mentioned in between the two potential antecedents and the anaphor with the aim of drawing participants' gaze away from the images of the potential antecedents before the onset of the target anaphor. While this manipulation did lead to at least twice as many looks to the target inanimate object image as to other images, the amounts of looks to the two potential antecedent images were not equal at the onset of the anaphor. Figure 5 contains graphs of the proportion of looks to the accessible antecedent and the inaccessible antecedent image in the single match and the double match condition for reflexives and pronouns starting at 600 ms before the onset of the target anaphor. In Figure 5A, at 600 ms before the onset (–600 ms) of the reflexive, the proportion of looks to the accessible antecedent is much higher than the proportion of looks to the inaccessible antecedent, and is decreasing until 300 ms after the onset, in both the double match and the single match condition. In contrast, in Figure 5B, a reversed pattern of looks is attested in the same time period for the pronoun, with the proportion of looks to the inaccessible antecedent image starting much higher and decreasing over time. This pattern of looks cannot be a function of the target anaphor as the pattern emerges much before the onset of the pronoun or the reflexive, and so it must have been driven by the co-argument subject of the clause, which corresponds to the accessible antecedent for the reflexive and the inaccessible antecedent for the pronoun. Hence, we cannot conclude that the timing of Principle B effects is later than Principle A effects based on the asymmetric pattern of looks between reflexives and pronouns attested in the early time period. In fact, in Figures 3B, 5B, it can be seen that at about 150 ms after the onset of the pronoun, the proportions of looks to the accessible antecedent in both the single match and the double match condition are starting to gradually increase. As a reviewer points out, changes in looks at this point may be too early to be in response to the pronoun. However, the trajectory of looks to the accessible antecedent at this point is suggestive of a beginning of an engagement of Principle B in pronoun processing. The same reviewer notes that the results in the 301–600 ms time period are confounded with the effects from looks to the most recently mentioned person, and that this makes it difficult to disentangle the effects of recency and the effects of reflexive/pronoun interpretation. While we completely acknowledge this difficulty, it is worth observing that even though this recency effect in pronouns resulted in more looks to the inaccessible antecedent, working against the effects of Binding Principle B in the early time period, it did not result in a significantly lower Binding Theory score for pronouns in comparison to reflexives, and after 600 ms, it did not result in a higher amount of looks to the inaccessible antecedent for pronouns in comparison to reflexives. We thus maintain our interpretation of the results that Principle B is engaged in early processing. As for Principle A, as there are already more looks to the accessible antecedent than the inaccessible antecedent for reflexives in the early time period, we have no evidence against early engagement of Principle A. Some hint of evidence for the early engagement of Principle A can be observed in Figures 3A, 5A in that the proportion of looks to the accessible antecedent is decreasing until 300 ms, and starts to increase at around 500 ms. This pattern of looks could be interpreted as a result of the Condition A effect surpassing the recency effect in the 301–600 ms time period.

A reviewer suggests that another possibility for the lower Binding Theory score for pronouns in the early time period is that the comprehenders might have to first rule out the possibility of co-reference before attempting binding. In linguistic theory, it has been widely posited that pronominal resolution can be achieved via binding that is subject to structural constraints, or co-reference that relies on discourse-based cues or morphological cues such as feature matching. However, it is not clear whether the processor prefers to apply co-reference interpretation before binding interpretation, or vice versa. While some authors have argued that binding is preferred over co-reference (Grodzinsky and Reinhart, 1993; Koornneef, 2008), others have shown that the preference for binding or co-reference is determined by factors such as prominence or recency of the potential antecedent (Frazier and Clifton, 2000; Cunnings et al., 2014). Perhaps the mere fact that pronoun interpretation is subject to ambiguity is a contributing factor to the lower Binding Theory score. It is certainly possible that the pronoun in the single match condition in Experiment 2 could be interpreted via binding through engaging structural constraints, or via co-reference through applying feature matching. But, under this reasoning, we would expect a higher Binding Theory score in the double match condition in comparison to the single match condition for pronouns in the same time period. In the double match condition, pronouns should unambiguously use the binding strategy because structural constraints must be engaged in order to rule out the inaccessible antecedent. However, we found no difference between the Binding Theory scores of pronouns in the two match conditions.

**Figure 6:**
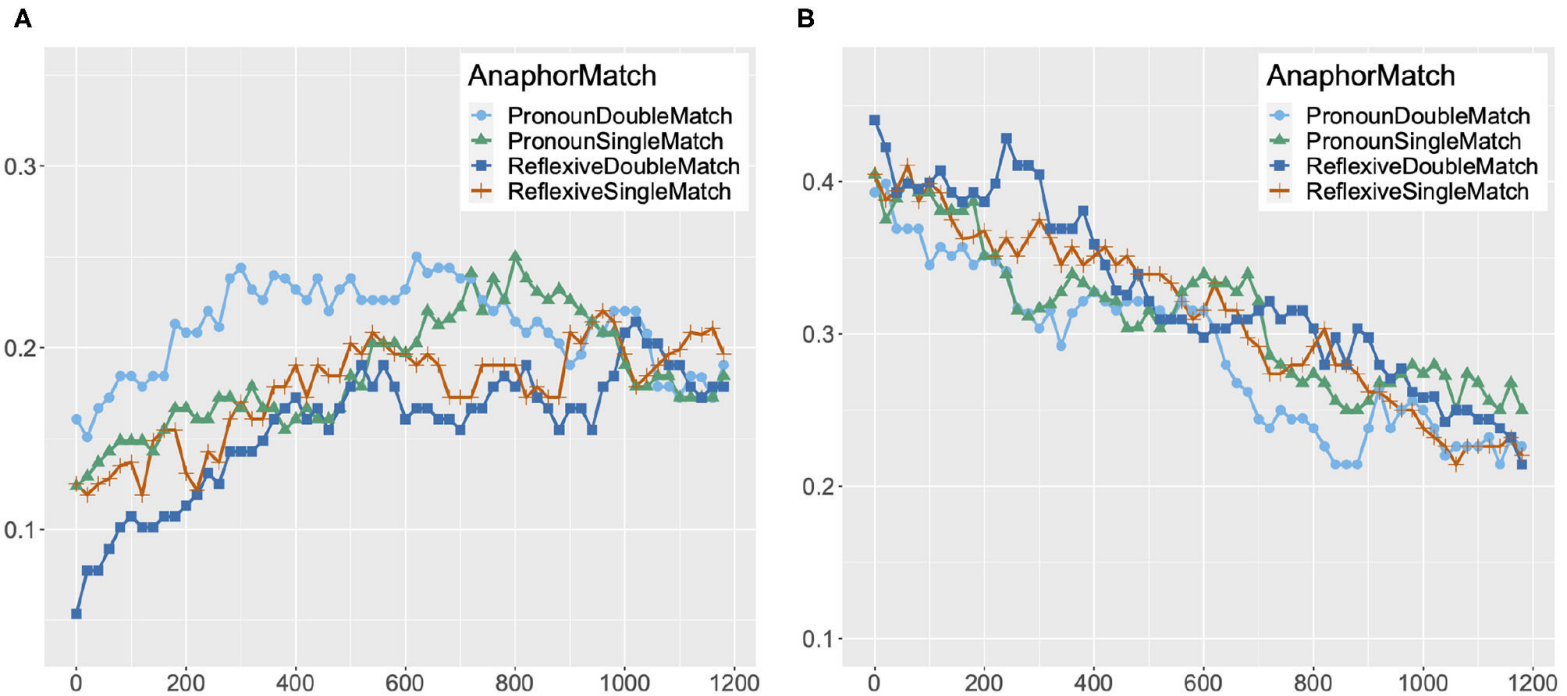
Proportion of looks to distractor and target object image in double/single match condition at every 20 ms from the onset of the target anaphor to 1,200 ms after the onset, Experiment 2. **(A)** Proportion of Looks to Distractor Object. **(B)** Proportion of Looks to Target Object.

In addition, as will be discussed in section 4, in a sentence completion task study, we found that native speakers of English predominantly treat the critical verbs we used in our trials as transitive and expect an inanimate theme argument as a direct object. It is thus very likely that our participants in Experiment 2 initially treated the verbs preceding the target anaphor as transitive as well. Then, this would have prompted the participants to shift their attention to a semantically compatible inanimate object during the presentation of the critical verb, resulting in anticipatory looks to the potential object (e.g., Altmann and Kamide, 1999). Note that our display in Experiment 2 had two inanimate object images, a target object mentioned in the sentence immediately before the verb, and a distractor object, not mentioned in the sentence. As can be seen in Figure 6A, the proportion of looks to the distractor object starts to increase at the onset of the target anaphor. Also, Figure 6B shows that the proportion of looks to the target object is high at the onset, which is expected as the expression referring to the target object has been presented just before the critical verb and the target anaphor [e.g., *a bucket* in (14)]. Furthermore, the proportion of looks to the target object remains high, only gradually decreasing throughout the plotted time period. This means that for pronouns, both the fact that the co-argument subject corresponds to the inaccessible antecedent and the likelihood that the verb preceding the pronoun is analyzed as a transitive verb requiring an inanimate object may have conspired to delay the looks to the accessible antecedent. On the other hand, this delayed effect may not have been so pronounced for reflexives, as their co-argument subject corresponds to the accessible antecedent.

**Figure 7:**
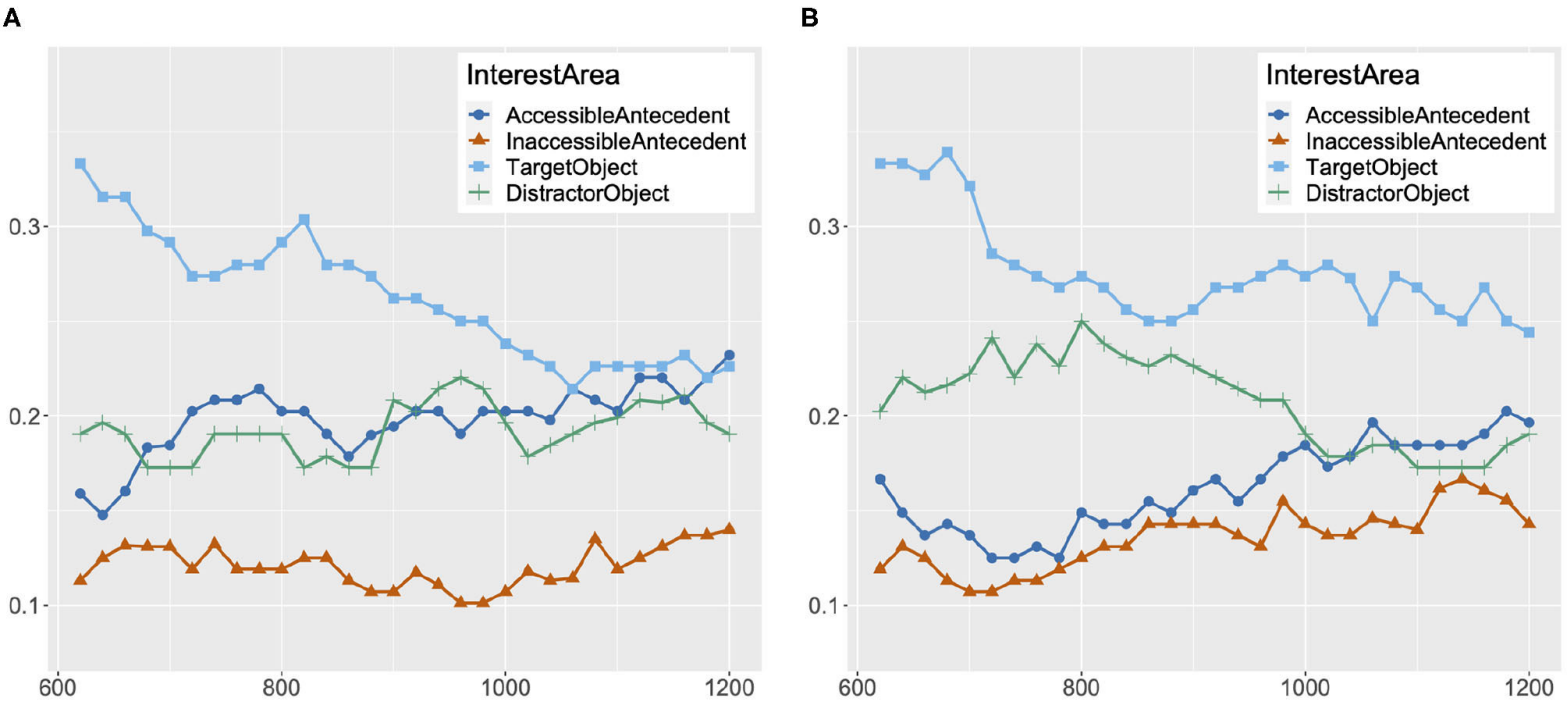
Proportion of looks to accessible antecedent, inaccessible antecedent, distractor object and target object image in single match condition at every 20 ms from 601 to 1,200 ms after the onset of the target anaphor, Experiment 2. **(A)** Proportion of Looks: Reflexive, Single Match. **(B)** Proportion of Looks: Pronoun, Single Match.

In sum, the results of all measures of the VWP eye-movement data in Experiment 2 support the hypothesis that the Principle B effect on pronouns is no weaker than the Principle A effect on reflexives in anaphoric processing. However, an unexpected finding was that when there was only one gender-matched accessible antecedent, pronouns showed a reduction in looks to the accessible antecedent image in the 601–900 and 901–1,200 ms time periods. This is evident in the graphs in Figure 7, which plot the proportion of looks to the accessible antecedent image, the inaccessible antecedent image, the target object image and the distractor object image for pronouns and reflexives in the single match condition from 601 to 1,200 ms after the onset of the anaphor. The graphs show that pronouns have a reduced proportion of looks to the accessible antecedent image in comparison to reflexives after 600 ms. This coincides with more looks to the distractor object image in 601–900 ms, and more looks to the target object image after 900 ms for pronouns. Comparisons between the fixation data for pronouns and reflexives, by means of generalized linear mixed-effects models, revealed that pronouns had marginally more looks to the distractor object image than reflexives in 601–900 ms (Estimate = 0.05, *SE* = 0.03, *z* = 1.85, *p* = 0.06), and that pronouns had significantly more looks to the target object image than reflexives in 901–1,200 ms (Estimate = 0.06, *SE* = 0.03, *z* = 2.22, *p* = 0.03). It is unclear why images other than the accessible antecedent image were fixated more frequently during this time period for pronouns in comparison to reflexives in the single match condition. Pronouns are single-syllabled with weak stress as opposed to reflexives that are multi-syllabled with more prominent prosody. Perhaps, in an unambiguous context with one clear possible antecedent, this difference in prosody enabled some participants to more easily shift their attention away from the referents of pronouns as opposed to those of reflexives.

Another possibility is that the fixation results may be confounded with the discourse status of the anaphor. Note that in the material in the single match condition in (14b), the subject of the sentence, which is the accessible antecedent for the target pronoun *her*, is also a pronoun (*she*). Pronouns encode different discourse status of their referents than referring expressions such as *the old man*. For example, in the Givenness Hierarchy framework proposed in Gundel et al. (1993), a pronoun is felicitously used when the speaker can assume that the addressee has their attention focused on its referent. In the single match condition in (14b), *she* implies, for the first time in the sentence, that the comprehender's attention is focused on its referent, the young girl. In the pronoun version of (14b), this referent is referred to by another pronoun *her* downstream. Thus, the comprehender's attention continues to be focused on the young girl, the only possible referent of *her* in the single match condition. It may be that in a non-ambiguous discourse context where the only possible referent of a pronoun is continuously in focus of attention, the reference tracking of the pronoun in question does not necessarily register as fixations in VWP. On the other hand, in the reflexive version of (14b), *himself* does not refer to the referent that is in focus of attention, the young girl. Instead, it refers to the referent expressed by the referring expression *the old man*, which is used felicitously, according to Gundel et al. (1993), when its conceptual content can enable the addressee to associate a unique representation with the expression. Thus, this is not a discourse context where the target referent is continuously in focus of attention. Moreover, in the double match condition in (14a), although the target pronoun *him* indicates that its referent continues to be in focus of attention, it is used in an ambiguous discourse context with two feature matching potential antecedents. In such discourse contexts, the need to disambiguate may translate to more fixations on the target referent in comparison to non-ambiguous discourse contexts. Testing this possible explanation by redoing the current VWP study using materials that contain target anaphors and antecedents with the exact same discourse status across conditions will have to wait for future work.

## 4. Experiment 3: Sentence Completion

Previous VWP eye-tracking studies have shown that listeners shift their attention to semantically compatible direct objects during the presentation of a transitive verb (e.g., Altmann and Kamide, 1999). While we are interested in the processing of verb-adjacent indirect arguments (a recipient or beneficiary of the action), we recognize that listeners are likely to expect a direct (theme) argument directly following the verb. In order to understand the strength of this likely expectation, we conducted a sentence completion task. We also wanted to ensure that if there is a bias to expect a direct (theme) argument following the verb, the strength of the bias should be equal across the test conditions (Double match vs. Single match). In this task, participants were presented with our experimental sentences up to and including the critical verb, and were asked to fill in the likely next word(s) of the sentence.

### 4.1. Methods

#### 4.1.1. Participants

Thirteen native English speakers were recruited from the university community, none of whom had participated in Experiment 1 or Experiment 2. Course credit was received upon completion of the experiment.

#### 4.1.2. Task, Design, and Materials

Participants performed a sentence completion task on a total of 48 items: 24 test items and 24 filler items. Each item was introduced by a general context sentence (16a) followed by a pair of sentences introducing two characters [(16b) or (16c)]. Test items were adapted from those used in Experiment 1 and Experiment 2, but designed with only one factor (Match) of two levels: Double match, in which the two characters are of the same gender; and Single match, in which the two characters are of different genders. The target sentence for each item was provided up to the verb head of the embedded clause [*built* in (16b) and (16c)]. Each verb had the potential to be interpreted as either a transitive verb or a ditransitive verb. This allowed participants to complete the verb phrase as either a single or a double object construct. All test items were distributed over two lists in a Latin-square design.

(16) Test Items
  - Context
    It was the first day of Summer vacation.
  - Double match
    The young boy was spending a day at the beach. He was amazed to see that the old man who was carrying a bucket had built _______________
  - Single match
    The young girl was spending a day at the beach. She was amazed to see that the old man who was carrying a bucket had built _______________

Each of the 24 filler items was comprised of one general context sentence followed by an incomplete target sentence. The target sentence was provided only up until the verb head, which was either unambiguously transitive (17a) or intransitive (17b). In all filler items, target sentences followed the same format: PP, the subject with a relative clause, and the verb head. Unlike the test items, only one character was introduced with each filler item. These filler items were added to each of the two lists.

(17) Filler items
  - Transitive
    It was a very cold day in December. In the living room, the old woman who was sitting by the fireplace avoided _______________
  - Intransitive
    It was a hot summer day in August. At the swimming pool, the young boy who was wearing a hat sneezed _______________

#### 4.1.3. Procedure

Participants were directed to the online experiment platform PennController for IBEX (Zehr and Schwartz, 2018). They received instructions to complete each unfinished sentence by adding at least one word, and completed two practice trials before proceeding with the experimental trials. After completing the experiment, participants filled out a brief demographic survey.

### 4.2. Results

The participants' interpretation of the argument structure of the verb in each trial was categorized into intransitive, prepositional dative, double object, and transitive, based on the expression they provided to complete the given sentence. The expression was an adjunct in intransitives (18a), a theme nominal argument followed by a prepositional argument in prepositional datives (18b), a recipient nominal argument followed by a theme nominal argument in double objects (18c), and a theme nominal argument in transitives (18d). In addition, the animacy of the expression was categorized into animate or inanimate. In cases where two arguments were provided, only the first argument was categorized for animacy.

(18)
  - She smiled when the young boy who was wearing thick socks knit by the fireplace.
  - He wondered why the young boy who was wearing a dress shirt sent a package to Florida.
  - He wondered whether the old man who was reading a magazine had bought him a new set of flippers.
  - He was amazed to see that the old man who was carrying a bucket had built a sandcastle.

**Figure 8:**
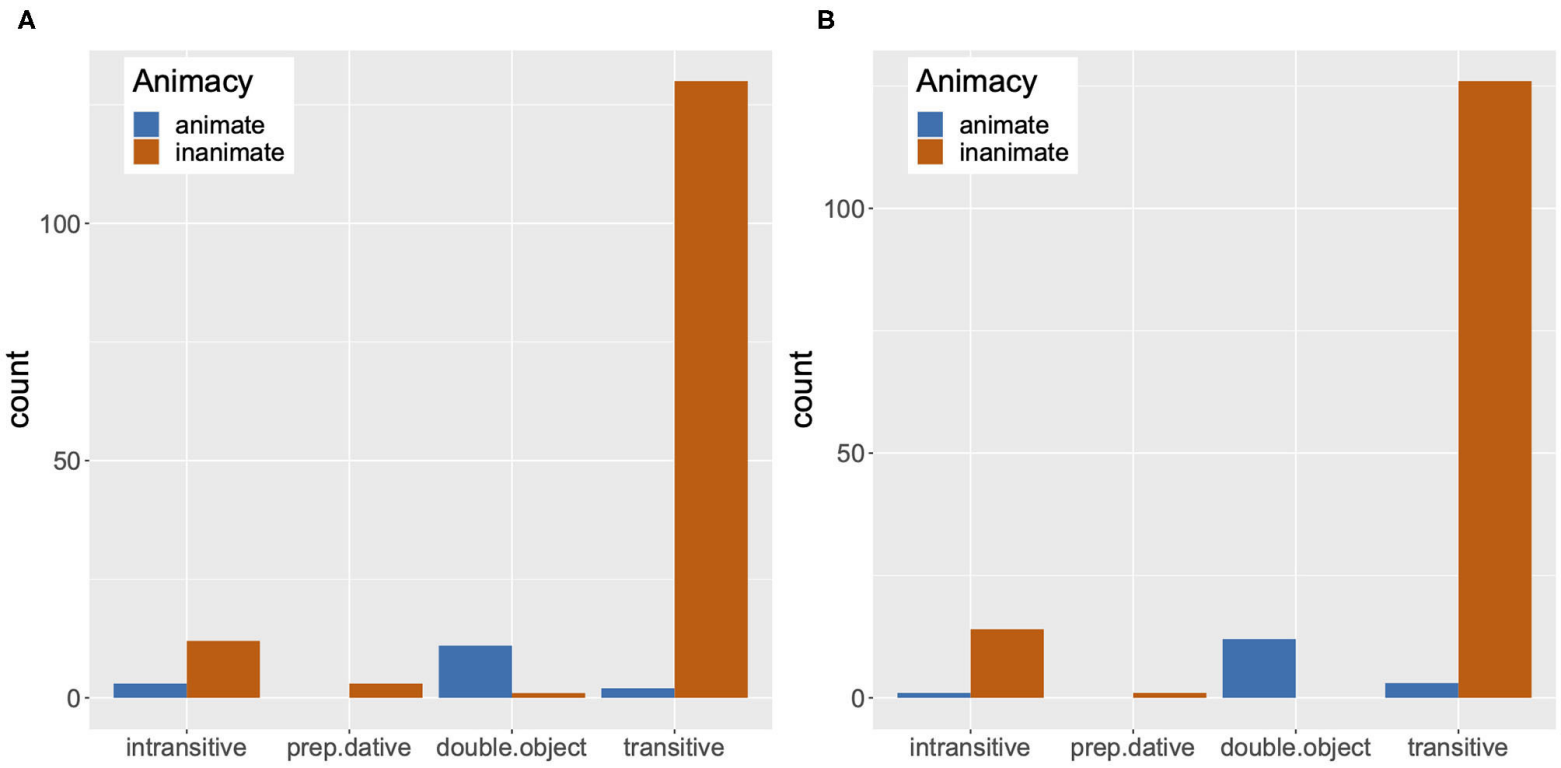
Frequency counts of argument structures grouped by animacy for the expressions provided by participants in the single/double match condition, Experiment 3. **(A)** Double Match. **(B)** Single Match.

The frequency counts of the argument structures are visualized in Figure 8, grouped by animacy for the expressions provided by the participants in the double match and the single match conditions. In both conditions, the most frequent argument structure was transitive with an inanimate theme argument. There were a few instances of double object structures, in which case the recipient argument was predominantly animate.

**Figure 9:**
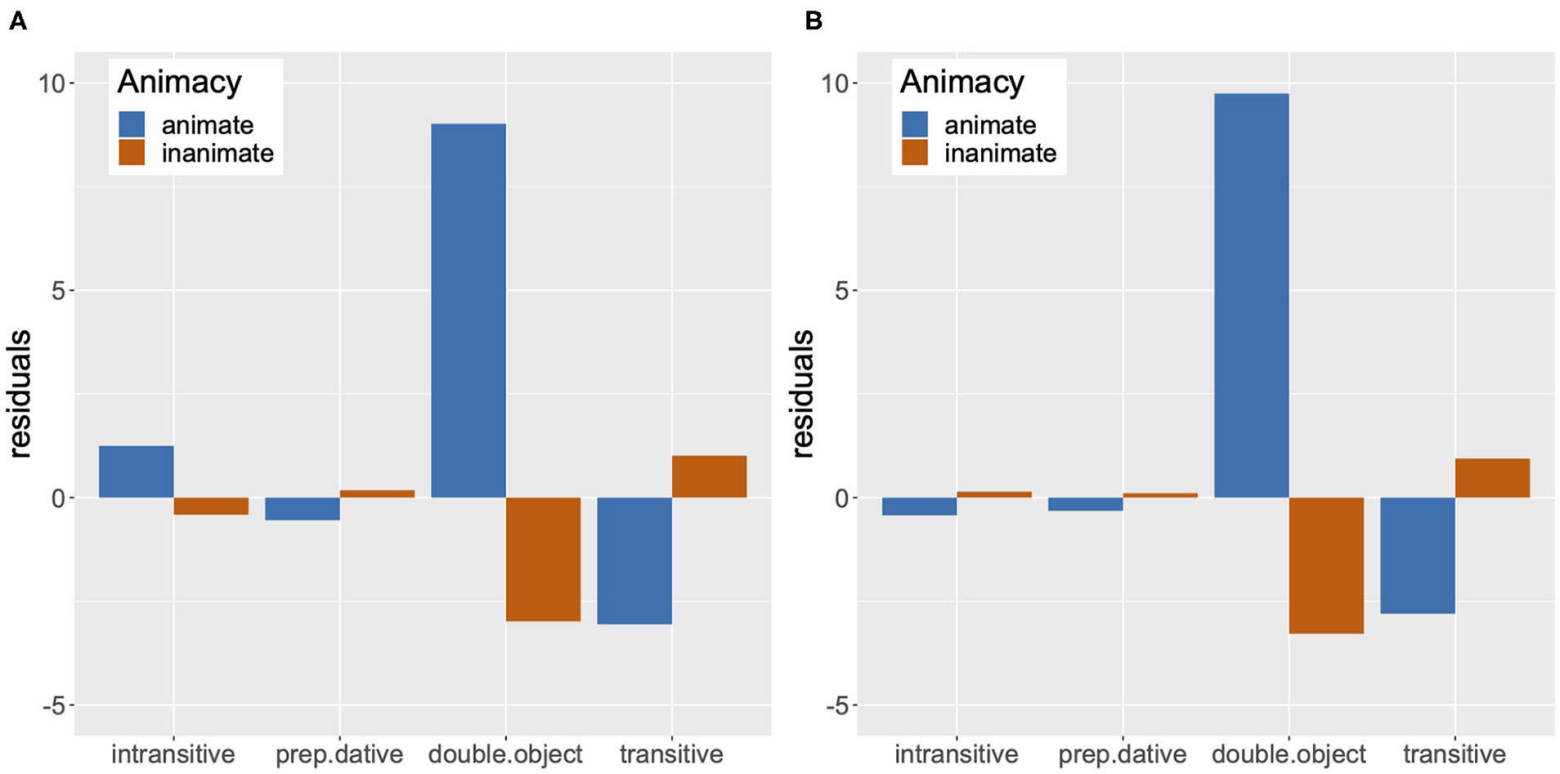
Pearson residuals of each argument structure grouped by animacy for the expressions provided by participants in the single/double match condition, Experiment 3. **(A)** Double Match. **(B)** Single Match.

A chi-squared test revealed that overall, the argument structures were significantly not evenly distributed (χ^2^ = 553.89, df = 3, *p* < 0.001), with the double match and the single match conditions exhibiting a similar shape of distribution of the argument structures (χ^2^ = 0.96, df = 3, *p* = 0.81). Moreover, Pearson's chi-squared tests revealed that argument structure and animacy were significantly associated in both the double match condition (χ^2^ = 102.61, df = 3, *p* < 0.001) and the single match condition (χ^2^ = 114.79, df = 3, *p* < 0.001). Pearson residuals of each argument structure grouped by animacy for the expressions provided by the participants in the single and the double match conditions are visualized in Figure 9 to show the direction of the association between argument structure and animacy. In both conditions, it can be seen that the double object structure is positively associated with an animate argument and negatively associated with an inanimate argument, whereas the transitive structure is positively associated with an inanimate argument and negatively associated with an animate argument. These results suggest that in both the double match and the single match condition, if the critical verb is interpreted as a double object verb, it is likely to be continued with an animate argument, and if the critical verb is interpreted as a transitive verb, it is likely to be continued with an inanimate argument. Moreover, as the critical verb is most likely to be interpreted as a transitive verb, the animacy of the expression provided by the participants is most likely to be inanimate.

In filler trials, the unambiguously transitive verbs were predominantly interpreted as transitive, and the unambiguously intransitive verbs were predominantly interpreted as intransitive, according to the responses provided to complete the target sentences. The participants' performance on filler trials is as expected, and thus lends support to the validity of the results on the test items.

### 4.3. Discussion

The findings of Experiment 3 suggest that the participants in Experiment 2 were likely to initially interpret the critical verbs as transitive and expect an inanimate theme argument immediately following the verb. This would have resulted in increased anticipatory looks to the potential inanimate object, delaying or possibly even reducing the looks to the target animate image. We have already seen this to be the case in Experiment 2 in Figure 6. But crucially, we found that the extent to which an inanimate theme argument was expected was the same in both the double match and the single match condition. Therefore, the amount of anticipatory looks to the potential object should have been the same across test conditions. To test this prediction, we analyzed the looks to the target or the distractor object image from the onset of the critical anaphor up to 600 ms in Experiment 2, by means of a generalized linear mixed-effects model. The model was fit to the data with fixed effects of Bin Index (for each 20 ms time period), Anaphor (Pronoun vs. Reflexive) and Match (Single match vs. Double match). Random-effects structure was included, and predictor variables were sum-coded, as described in section 3.2.2. The analysis revealed an interaction of Anaphor and Match (Estimate = 0.04, *SE* = 0.01, *z* = 3.53, *p* < 0.001). However, upon planned comparisons between the fixation data for pronouns and reflexives, we found no significant difference between the single match and the double match condition for either anaphor type (Pronoun: Estimate = 0.09, *SE* = 0.09, *z* = 1.00, *p* = 0.32; Reflexive: Estimate = –0.02, *SE* = 0.08, *z* = –0.23, *p* = 0.82). Looking at the estimates and the *z* values, the interaction seems to be due to a slightly higher amount of looks to a (target or distractor) object image in the double match condition than in the single match condition for pronouns and the reverse pattern of looks for reflexives. Therefore, in Experiment 2, the overall amount of anticipatory looks to the potential object (distractor or target object image) is not much different across test conditions, and thus our interpretation of the main findings from Experiment 2 is not affected.

## 5. General Discussion

Our goal in this paper was to investigate the strength of Principle B effects on pronoun processing, in comparison to the strength of Principle A effects on reflexive processing, when pronouns and reflexives occur in an argument position, an environment properly subject to Binding Theory (Reinhart and Reuland, 1993). We considered two hypotheses: (i) the Principle B effect on pronouns is as strong as the Principle A effect on reflexives, and (ii) the Principle B effect on pronouns is weaker than the Principle A effect on reflexives. If the Principle B effect is as strong as the Principle A effect in anaphoric processing, the amount of interference from a gender-matched but structurally inaccessible antecedent should be the same across pronouns and reflexives. But if the Principle B effect is weaker than the Principle A effect, pronouns should be more susceptible to interference from an inaccessible antecedent than reflexives.

In our VWP eye-tracking study (Experiment 2), we found that the extent to which comprehenders consider a structurally accessible antecedent over a gender-matched but structurally inaccessible antecedent is similar in both pronoun and reflexive processing. This result suggests that the amount of interference from a gender-matched inaccessible antecedent is the same across pronouns and reflexives, and supports the hypothesis that the strength of the Principle B effect is equal to the strength of the Principle A effect when applied to anaphors in argument positions. However, we unexpectedly found a reduced amount of looks to the accessible antecedent for pronouns in the single match condition after 600 ms in comparison to the pronouns in the double match condition and the reflexives in the single/double match condition. Importantly, this did not result in a higher amount of looks to the inaccessible antecedent in comparison to other conditions during this time period, suggesting that the interference from the gender-mismatched inaccessible antecedent for pronouns is no greater than the interference from the gender-matched inaccessible antecedent.

In Experiment 1, we saw that while our participants generally made off-line judgments in accordance with the Binding Principles, they sometimes erroneously selected a structurally inaccessible antecedent when it matched in gender with the critical anaphor. But crucially, the extent to which an inaccessible antecedent was selected over the accessible antecedent was the same for both pronouns and reflexives. Thus, these results affirm that the strength of the Principle B effect on pronouns is no different from the strength of the Principle A effect on reflexives in the off-line judgments. Moreover, in Experiment 1, the selection of a structurally accessible antecedent for pronouns in the single match condition was as secure as for the reflexives in the same condition. This is different from the on-line behavior where we found that the amount of looks to a structurally accessible antecedent for the pronouns was reduced in comparison to the reflexives in the single match condition. Hence, the apparent difficulty detected with the pronoun resolution in the single match condition in the on-line behavior is non-existent in the off-line judgments.

Our data also suggest that the structural constraints of Binding Theory are applied at the early stages of anaphoric processing for both pronouns and reflexives. As can be seen in Figure 3, for reflexives, the amount of looks to the accessible antecedent image is already higher than the amount of looks to the inaccessible antecedent image at the onset of the anaphor, due to a delayed response to the co-argument subject, but continues to remain high before increasing even further. For pronouns, although the amount of looks to the accessible antecedent image is lower than the amount of looks to the inaccessible antecedent image at the onset, again due to a delayed response to the co-argument subject, it starts to increase soon after the onset, eventually surpassing the amount of looks to the inaccessible antecedent image. These patterns of looks suggest that Binding Theory is engaged at this early stage by the comprehenders in interpreting anaphors. At the same time, structurally inaccessible antecedents are also being considered for both pronouns and reflexives, as can be seen by the presence of some amount of looks to inaccessible antecedent images throughout the analyzed time period. However, structurally inaccessible antecedents are considered no more than structurally accessible antecedents, and the degree to which gender-matched inaccessible antecedents are considered is no more than the baseline degree to which gender-mismatched inaccessible antecedents are considered.

Overall, our findings are not fully compatible with the Binding-as-initial-filter model (Nicol and Swinney, 1989; Clifton et al., 1997) nor a strong version of Chow et al.'s (2014) *Simultaneous Constraints* hypothesis, in which both structural constraints and morphological cues are applied without exception so that neither structurally inappropriate nor morphologically non-matching antecedents are retrieved. If looks to the inaccessible antecedent in the VWP are evidence of retrieval, then the fact that both pronouns and reflexives trigger some number of looks to such antecedents suggests that structural constraints do not entirely govern the process. Instead our results comport with what Chow et al. (2014) describe as a weakening of the Simultaneous Constraints hypothesis, whereby structural constraints interact with other cues. However, we found no evidence that structural constraints can be outweighed by non-structural considerations. Recall that for both pronouns and reflexives, in none of the time periods analyzed were inaccessible antecedents considered significantly more than accessible antecedents.

Our results are also compatible with the suggestion in Parker and Phillips (2017) and Jäger et al. (2020) that structural constraints have a higher weight than non-structural ones but that the weighting of non-structural cues can be greater than zero. However, the morphological gender cue on inaccessible antecedents proved not to play a significant role in our results. For both pronouns and reflexives, the inaccessible antecedent with matching gender did not elicit more looks than the non-matching inaccessible antecedent. In their eye-tracking while reading study, however, Jäger et al. (2020) did find an inhibitory interference effect for reflexives in early reading measures: more first pass regressions from reflexives in double match conditions than single match conditions. This kind of processing cost is an expected consequence of cue-based retrieval models (see Lewis and Vasishth, 2005): when multiple items match a retrieval cue, this increases retrieval time. We might have expected that such a multiple match effect would show up in the VWP as a tighter competition in the proportion of looks between accessible and inaccessible referents in the double match condition compared to the single match condition. The lack of a double match effect in our VWP data does not necessarily mean that there is no interference at all from inaccessible antecedents. There were still some amount of looks to both the gender-matched and the gender-mismatched inaccessible antecedents. It is arguable that these looks are due to some morphological retrieval cues (such as animacy, number, or syntactic category) that are weighted much lower than structural constraints but higher than gender cues. There may be several reasons why we did not observe a double match effect in the VWP even with a non-zero weight on gender cues. Statistical power may be an issue (see section 3.1.1): greater power might reveal more looks to the gender-matching inaccessible antecedent than the non-matching one. Another possibility is that the increase in retrieval time associated with multiple match configurations does not align straightforwardly with measures of attention in the VWP. That is, the division of looks may not correlate directly with levels of activation that lead to processing delays; having the target referent in the visual field in the VWP paradigm, and not in just memory as in reading paradigms, may influence looks in the interpretation of anaphors. Further experimentation and modeling is needed to relate the processing costs of inhibitory interference to the mechanisms that underlie visual attention in the VWP (see Sekerina et al., 2016).

Lastly, our results on pronouns are consistent with those found by Chow et al. (2014), who did not find evidence of inhibitory interference for pronouns. As with reflexives, we found no difference in the amount of looks to the matching and non-matching inaccessible pronouns (i.e., no double match effect). However, given Jäger et al.'s (2020) findings for reflexives and the fact that we did not find the relevant differences between pronouns and reflexives, Chow et al.'s (2014) results on pronouns remain outstanding. Here again the issue of power may be relevant [Chow et al.'s (2014) eye-tracking while reading study employed 24 participants compared to Jäger et al.'s (2020) 190].

## 6. Conclusion

In the study reported in this paper, we investigated whether structural constraints (Binding Principle A for reflexives and Binding Principle B for pronouns) are equally engaged in the early stages of anaphoric processing. Based on the findings from our VWP eye-movement data, we argue that when anaphors are in argument positions, pronouns are just as sensitive to Principle B as reflexives are to Principle A in early processing.

## Data Availability Statement

The raw data supporting the conclusions of this article will be made available by the authors, without undue reservation.

## Ethics Statement

The studies involving human participants were reviewed and approved by Simon Fraser University, Office of Research Ethics. The patients/participants provided their written informed consent to participate in this study.

## Author Contributions

C-hH and KM designed the experiments. TB, HG, and SN programmed the experiments and collected data. C-hH performed statistical analysis. C-hH, KM, TB, HG, and SN wrote the paper. All authors contributed to the article and approved the submitted version.

## Conflict of Interest

The authors declare that the research was conducted in the absence of any commercial or financial relationships that could be construed as a potential conflict of interest.

## Footnotes

^1^Another VWP study that investigates looks to inaccessible antecedents for reflexives and pronouns is reported in Choy and Thompson (2010). Unlike Clackson et al. (2011), Choy and Thompson (2010) put the reflexive and pronoun in a direct object position. Their study, however, was focused on aphasic participants, with only eight control participants. Moreover, they do not include a single match condition to serve as a baseline for looks to the matrix subject referent. As a result, their results do not provide a measure for how much a matching, interfering referent steals looks from the accessible antecedent. Furthermore, the critical animate noun phrases used in their stimuli are not consistently gendered.

^2^Clackson and Heyer (2014) report on a VWP study that tested the double match effect on reflexive processing using the same set of stimuli as in Clackson et al. (2011). Unlike the task in the Clackson et al. (2011) study, the task in the Clackson and Heyer (2014) study was goal-directed: the participants were required to identify the referent of the reflexive at the end of each trial. They found a double match effect in that the rate at which the amount of looks to the accessible antecedent rose was slower in the double match condition than in the single match condition in the early time period (200–600 ms after the onset of the reflexive). As the critical reflexive in the stimuli in the Clackson and Heyer (2014) study was always located within a PP, a domain that does not neatly conform to Binding Theory for independent reasons, the question of a possible confound that we raised for the stimuli in the Clackson et al. (2011) study applies here as well.

^3^A reviewer notes that the sample size of 19 participants is too small, and this is possibly the reason why the statistical models with random slopes failed to converge. The sample size was based on the finding reported in Schütze and Sprouse (2014) that forced-choice tasks require a relatively small sample size (*N* = 11) to detect a difference between conditions with 80% power, in comparison to other tasks such as magnitude estimation or numerical rating tasks.

^4^We compared models with and without the fixed effect term Match with a likelihood ratio test using anova(). This revealed that Match significantly improves the fit of the model (χ^2^ = 23.53, df = 1, *p* < 0.001). Hence, there is a significant effect of Match. The likelihood ratio tests also showed that the fixed effect term Anaphor or the interaction term Match×Anaphor does not significantly improve the fit of the model.

^5^A reviewer notes that according to the power estimates reported by Jäger et al. (2020), a sample size of 28 participants does not yield sufficient statistical power to accurately measure interference effects. The same reviewer also notes that according to Vasishth et al. (2018), low power studies increase the chance of a type-M(agnitude) error where a significant result is due to an overestimate of the true effect. However, the findings reported in Jäger et al. (2020) and Vasishth et al. (2018) are based on eye-tracking while reading studies. Sample sizes in VWP studies are typically smaller: the VWP study in Clackson et al. (2011) tested 40 participants, the VWP study in Runner and Head (2014) tested 25 participants and in Kaiser et al. (2009), one VWP study tested 24 participants and two VWP studies tested 16 participants each. Nonetheless, we acknowledge that it would be ideal to replicate the findings reported here with higher power. We leave this for future work.

^6^A reviewer notes that the point where the reflexive is distinguished from the pronoun is different between the double match and the single match condition: that is, while the difference between *her* and *himself* (in the single match condition) is immediate, the difference between *him* and *himself* (in the double match) occurs a bit later, as the initial syllables of the two forms are the same. One might then expect this timing difference in pronoun/reflexive distinction to lead to a delayed effect of structural constraints for reflexives in the double match condition, in comparison to the reflexives in the single match condition. However, it is observed in Kaiser et al. (2009) that the durations of the free-standing pronouns *him* and *her* are significantly longer than the first syllables of the reflexives *himself* and *herself*, respectively. If free-stranding pronouns and the first syllables of reflexives are phonetically distinct, then the timing of pronoun/reflexive distinction in the double match condition may not be so different from the single match condition. In fact, in our eye-movement data, a differentiation between accessible and inaccessible antecedents, an effect of structural constraints, occurs no later in the double match condition than in the single match condition for reflexives. This suggests that even if the timing of the pronoun/reflexive distinction is late in the double match, this did not lead to a delayed response for reflexives.

^7^A likelihood ratio test using anova() showed that the fixed effect term Antecedent significantly improves the fit of the model (χ^2^ = 5.88, df = 1, *p* < 0.05). Hence, there is a significant effect of Antecedent. The likelihood ratio tests also showed that the fixed effect term Match or the interaction term Antecedent×Match does not significantly improve the fit of the model.

^8^A likelihood ratio test using anova() showed that the interaction term Antecedent×Match significantly improves the fit of the model (χ^2^ = 8.20, df = 3, *p* < 0.05). Hence, there is a significant interaction of Antecedent and Match.

^9^A series of likelihood ratio test using anova() showed that the fixed effect term Antecedent (χ^2^ = 5.02, df = 1, *p* < 0.05), the interaction term Antecedent×Anaphor (χ^2^ = 94.72, df = 3, *p* < 0.001), and the interaction term Antecedent×Match×Anaphor (χ^2^ = 115.21, df = 7, *p* < 0.001) each significantly improves the fit of the model.
