# Supplementary material for: Pronouns Are as Sensitive to Structural Constraints as Reflexives in Early Processing: Evidence From Visual World Paradigm Eye-Tracking
Source: Front Psychol. 2021 Feb 5;12:611466. doi: 10.3389/fpsyg.2021.611466 (PMC7893137; doi:10.3389/fpsyg.2021.611466)
Supplement: Supplementary file 1 [file Data_Sheet_1.PDF]

Supplementary Material

The sentence judgment materials for Experiment 1, and the auditory materials for Experiment 2 are provided below. The sentence completion materials used for Experiment 3 were modified from below.

1. a. The young boy was waiting outside the corner shop. He watched as the old man who was wearing a hat bought himself/him a huge box of popcorn.
   b. The young girl was waiting outside the corner shop. She watched as the old man who was wearing a hat bought himself/her a huge box of popcorn.
2. a. The young boy was laying the dinner table. He asked whether the old man who was holding a cup could fetch himself/him a large clean plate.
   b. The young girl was laying the dinner table. She asked whether the old man who was holding a cup could fetch himself/her a large clean plate.
3. a. The young boy was spending a day at the beach. He was amazed to see that the old man who was carrying a bucket built himself/him a magnificent sand castle.
   b. The young girl was spending a day at the beach. She was amazed to see that the old man who was carrying a bucket built himself/her a magnificent sand castle.
4. a. The young boy was visiting a dairy farm. He watched as the old man who was sitting on a bench made himself/him some special rich cheese.
   b. The young girl was visiting a dairy farm. She watched as the old man who was sitting on a bench made himself/her some special rich cheese.
5. a. The young boy was very interested in water sports. He wondered whether the old man who was reading a letter bought himself/him a new canoe.
   b. The young girl was very interested in water sports. She wondered whether the old man who was reading a letter bought himself/her a new canoe.
6. a. The young boy was feeling a little peckish. He saw that the old man who was holding a rock cracked himself/him a huge walnut.
   b. The young girl was feeling a little peckish. She saw that the old man who was holding a rock cracked himself/her a huge walnut.
7. a. The young girl was sitting at the kitchen table. She watched as the old woman who was trying out a new mixer made herself/her a chocolate cake.
   b. The young boy was sitting at the kitchen table. He watched as the old woman who was trying out a new mixer made herself/him a chocolate cake.
8. a. The young girl was sitting by the swimming pool. She noticed that the old woman who was holding a purse bought herself/her a large ice cream cone.
   b. The young boy was sitting by the swimming pool. He noticed that the old woman who was holding a purse bought herself/him a large ice cream cone.
9. a. The young girl was feeling very hungry. She watched as the old woman who was holding a plate baked herself/her a large apple pie.
   b. The young boy was feeling very hungry. He watched as the old woman who was holding a plate baked herself/him a large apple pie.

10. a. The young girl watched the snow falling outside. She could hear that the old woman who was holding a shovel made herself/her a log fire.
    b. The young boy watched the snow falling outside. He could hear that the old woman who was holding a shovel made herself/him a log fire.
11. a. The old man was watching the customers in the post office. He wondered why the young boy who was wearing a dress shirt sent himself/him a small package.
    b. The old woman was watching the customers in the post office. She wondered why the young boy who was wearing a dress shirt sent himself/her a small package.
12. a. The old man was standing in the kitchen. He noticed that the young boy who was holding a pot boiled himself/him a large egg.
    b. The old woman was standing in the kitchen. She noticed that the young boy who was holding a pot boiled himself/her a large egg.
13. a. The old man was listening very hard. He knew that the young boy who was practicing the banjo found himself/him some new classical music pieces.
    b. The old woman was listening very hard. She knew that the young boy who was practicing the banjo found himself/her some new classical music pieces.
14. a. The old man was looking forward to Halloween. He knew that the young boy who was dressing up as a ghost carved himself/him a giant pumpkin.
    b. The old woman was looking forward to Halloween. She knew that the young boy who was dressing up as a ghost carved himself/her a giant pumpkin.
15. a. The old man was very nervous before the TV interview. He suddenly realized that the young boy who was wearing a tie got himself/him a microphone.
    b. The old woman was very nervous before the TV interview. She suddenly realized that the young boy who was wearing a tie got himself/her a microphone.
16. a. The old man knew that winter wasn't very far away. He smiled when the young boy who was wearing fuzzy slippers knit himself/him a long woolen scarf.
    b. The old woman knew that winter wasn't very far away. She smiled when the young boy who was wearing fuzzy slippers knit himself/her a long woolen scarf.
17. a. The old man was very tired. He barely noticed that the young boy who was wearing a dress shirt ran himself/him a hot bath.
    b. The old woman was very tired. She barely noticed that the young boy who was wearing a dress shirt ran himself/her a hot bath.
18. a. The old man was fascinated by technology. He was impressed when the young boy who was holding a drill built himself/him a working radio.
    b. The old woman was fascinated by technology. She was impressed when the young boy who was holding a drill built himself/her a working radio.
19. a. for The old woman was looking around the toy shop. She frowned when the young girl who was eating a popsicle bought herself/her a broken toy.
    b. The old man was looking around the toy shop. He frowned when the young girl who was eating a popsicle bought herself/him a broken toy.

20. a. The old woman used to teach geography. She was pleased to see that the young girl who was holding a note pad drew herself/her a large map.
    b. The old man used to teach geography. He was pleased to see that the young girl who was holding a note pad drew herself/him a large map.
21. a. The old woman is grim-looking. She was annoyed when the young girl who was swinging a wine bottle bought herself/her a rum cocktail.
    b. The old man is grim-looking. He was annoyed when the young girl who was swinging a wine bottle bought herself/him a rum cocktail.
22. a. The old woman was making some lemonade. She asked whether the young girl who was chilling in the pool could fetch herself/her a clean glass.
    b. The old man was making some lemonade. He asked whether the young girl who was chilling in the pool could fetch herself/him a clean glass.
23. a. The old woman couldn't stand the sight of blood. She was relieved when the young girl who was carrying a band-aid got herself/her some disinfectant.
    b. The old man couldn't stand the sight of blood. He was relieved when the young girl who was carrying a band-aid got herself/him some disinfectant.
24. a. The old woman is a fashion designer. She was pleased to see that the young girl who was wearing a smart dress made herself/her a beautiful belt.
    b. The old man is a fashion designer. He was pleased to see that the young girl who was wearing a smart dress made herself/him a beautiful belt.
